# Serum coding and non‐coding RNAs as biomarkers of NAFLD and fibrosis severity

**DOI:** 10.1111/liv.14167

**Published:** 2019-06-26

**Authors:** Stefania Di Mauro, Alessandra Scamporrino, Salvatore Petta, Francesca Urbano, Agnese Filippello, Marco Ragusa, Maria T. Di Martino, Francesca Scionti, Stefania Grimaudo, Rosaria M. Pipitone, Graziella Privitera, Antonino Di Pino, Roberto Scicali, Luca Valenti, Paola Dongiovanni, Anna Fracanzani, Agata M. Rabuazzo, Antonio Craxì, Michele Purrello, Francesco Purrello, Salvatore Piro

**Affiliations:** ^1^ Department of Clinical and Experimental Medicine, Internal Medicine, Garibaldi‐Nesima Hospital University of Catania Catania Italy; ^2^ Section of Gastroenterology, Di.Bi.M.I.S University of Palermo Palermo Italy; ^3^ Department of BioMedical Sciences and BioTechnology Section of Biology and Genetics Giovanni Sichel, Unit of Molecular, Genome and Complex Systems BioMedicine Catania Italy; ^4^ Oasi Research Institute - IRCCS Troina 94018 Italy; ^5^ Department of Experimental and Clinical Medicine Magna Graecia University Catanzaro Italy; ^6^ Translational Medicine University of Milan, Fondazione IRCCS Ca' Granda Pad Marangoni Milan Italy; ^7^ Department of Pathophysiology and Transplantation, Section of Internal Medicine University of Milan, Fondazione Ca' Granda IRCCS Ospedale Maggiore Policlinico Milan Italy

**Keywords:** fibrosis, liquid‐biopsy, NAFLD, NASH, RNAs

## Abstract

**Background & Aims:**

In patients with non‐alcoholic fatty liver disease (NAFLD), liver biopsy is the gold standard to detect non‐alcoholic steatohepatitis (NASH) and stage liver fibrosis. We aimed to identify differentially expressed mRNAs and non‐coding RNAs in serum samples of biopsy‐diagnosed mild and severe NAFLD patients with respect to controls and to each other.

**Methods:**

We first performed a whole transcriptome analysis through microarray (n = 12: four Control: CTRL; four mild NAFLD: NAS ≤ 4 F0; four severe NAFLD NAS ≥ 5 F3), followed by validation of selected transcripts through real‐time PCRs in an independent internal cohort of 88 subjects (63 NAFLD, 25 CTRL) and in an external cohort of 50 NAFLD patients. A similar analysis was also performed on liver biopsies and HepG2 cells exposed to oleate:palmitate or only palmitate (cellular model of NAFL/NASH) at intracellular/extracellular levels. Transcript correlation with histological/clinical data was also analysed.

**Results:**

We identified several differentially expressed coding/non‐coding RNAs in each group of the study cohort. We validated the up‐regulation of UBE2V1, BNIP3L mRNAs, RP11‐128N14.5 lncRNA, TGFB2/TGFB2‐OT1 coding/lncRNA in patients with NAS ≥ 5 (vs NAS ≤ 4) and the up‐regulation of HBA2 mRNA, TGFB2/TGFB2‐OT1 coding/lncRNA in patients with Fibrosis stages = 3‐4 (vs *F* = 0‐2). In in vitro models: UBE2V1, RP11‐128N14.5 and TGFB2/TGFB2‐OT1 had an increasing expression trend ranging from CTRL to oleate:palmitate or only palmitate‐treated cells both at intracellular and extracellular level, while BNIP3L was up‐regulated only at extracellular level. UBE2V1, RP11‐128N14.5, TGFB2/TGFB2‐OT1 and HBA2 up‐regulation was also observed at histological level. UBE2V1, RP11‐128N14.5, BNIP3L and TGFB2/TGFB2‐OT1 correlated with histological/biochemical data. Combinations of TGFB2/TGFB2‐OT1 + Fibrosis Index based on the four factors (FIB‐4) showed an Area Under the Curve (AUC) of 0.891 (*P* = 3.00E‐06) or TGFB2/TGFB2‐OT1 + Fibroscan (AUC = 0.892, *P* = 2.00E‐06) improved the detection of *F* = 3‐4 with respect to *F* = 0‐2 fibrosis stages.

**Conclusions:**

We identified specific serum coding/non‐coding RNA profiles in severe and mild NAFLD patients that possibly mirror the molecular mechanisms underlying NAFLD progression towards NASH/fibrosis. TGFB2/TGFB2‐OT1 detection improves FIB‐4/Fibroscan diagnostic performance for advanced fibrosis discrimination.

**Abbreviations**
ALT: Alanine aminotransferase
APRI: aspartate aminotransferase‐to‐platelet ratio index
AST: Aspartate aminotransferase
AUC: area under the curve
BMI: body mass index
Ct: threshold cycle
DE: differentially expressed
FC: fold change
FIB‐4: four factors‐based fibrosis index
lncRNA: long non‐coding RNA
LSM: liver stiffness measurement
mRNA: messenger RNA
NAFL: non‐alcoholic fatty liver
NAFLD: non‐alcoholic fatty liver disease
NAS: NAFLD activity score
NASH: non‐alcoholic steatohepatitis
ncRNA: non‐coding RNA
OA: oleate
PA: palmitate
ROC: receiver operating characteristics

**LAY SUMMARY**

- Liver biopsy still remains the gold standard for NAFLD diagnosis confirmation, distinction between simple steatosis and NASH, and fibrosis staging. No study has been performed regarding the circulating lncRNA and mRNA signatures as biomarkers in NAFLD patient serum.
- Our study suggests the use of coding and non‐coding RNA as non‐invasive biomarkers of NAFLD and fibrosis severity. Combination of coding/non‐coding RNA expression levels and clinical data could be conveniently applied as a diagnostic tool for non‐invasive screening of NAFLD patients according to disease severity.

## 1 BACKGROUND

Non‐alcoholic fatty liver disease (NAFLD) accounts for the most increasing cause of chronic liver disease, hepatocellular carcinoma and of end‐stage liver disease leading to liver transplantation.^1^, ^2^

Cohort studies demonstrated that among NAFLD populations, those with non‐alcoholic steatohepatitis (NASH) have a higher risk of fibrosis progression, and that the presence of severe liver fibrosis is the main driver of hepatic and extra‐hepatic prognosis.^2^, ^3^ Even if liver biopsy is considered the diagnostic gold standard, the availability of non‐invasive markers to be used in NAFLD patients to predict NASH and/or severity of fibrosis represents a relevant medical need.^4^ To date, the diagnosis of NASH in clinical practice by using non‐invasive scores is difficult because of the lack of well‐performing and well‐validated tools.^5^ Furthermore, novel‐proposed NASH blood biomarkers, such as CK18‐Asp396 fragments, showed low sensitivity for NASH detection.^5^, ^6^ Different scores are available for the non‐invasive assessment of fibrosis (eg ‘aspartate aminotransferase (AST)‐to‐platelet ratio index’ (APRI) and the ‘four factors‐based fibrosis index’ (FIB‐4); some of them are very easy to use and implement in clinical practice even if there are limitations such as false‐positive results and high uncertainty areas.^3^, ^7^ When looking at imaging devices, liver stiffness measurement (LSM) through transient elastography (FibroScan) is a widely diffused tool, even if its accuracy can be influenced by obesity and severity of steatosis.^7^, ^8^

Circulating RNAs in plasma or serum have been attracting exponential attention as novel non‐invasive diagnostic biomarkers of several kinds of diseases. Body fluid RNAs, including long non‐coding RNAs (lncRNAs) and messenger RNAs (mRNAs), have many of the essential characteristics of good biomarkers, for example: (i) they are non‐invasive accessible; (ii) their levels can easily be determined by basic molecular biology methods, first of all real‐time PCR; (iii) they are remarkably stable in spite of the high amounts of endogenous ribonuclease in body fluids such as serum and plasma.^9^, ^10^, ^11^

Several studies have highlighted the stability of lncRNAs and mRNAs, also under various experimental and pre‐analytical oppressive conditions, including multiple freeze‐thaw cycles, prolonged incubation at room temperature (up to 24h), exogenous RNAse treatments, time delay in processing of blood after venipuncture, low/high pH.^10^, ^12^, ^13^, ^14^, ^15^

The stability of lncRNAs and mRNAs in different body fluids against endogenous ribonuclease degradation may be provided by vesicles encapsulation and/or RNA binding protein association and also their highly stable secondary structures.^10^, ^11^, ^16^, ^17^

Two very recent studies reported the aberrant expression of long non‐coding RNAs (lncRNAs) in liver tissue of NAFL/NASH patients and in mice models of NAFLD.^18^, ^19^ Furthermore, increasing experimental evidence is beginning to characterize the role of specific lncRNAs in the pathogenesis of liver fibrosis 20 and several metabolic functions, such as free fatty acid β‐oxidation, lipogenesis and insulin secretion.^21^ Numerous studies performed high‐throughput analysis of mRNAs in liver tissues from NAFLD patients or in vivo animal models, which allowed the identification of several pathways associated with disease progression.^22^, ^23^, ^24^, ^25^, ^26^ To date, no study has been performed on circulating mRNA and lncRNA signatures in sera from NAFLD patients. Therefore, the main aim of this work was to analyse the whole transcriptome profile in serum samples of NAFLD biopsy‐diagnosed patients to identify novel non‐invasive biomarkers which are able to identify NAFLD patients with NASH and/or advanced fibrosis.

## 2 MATERIALS AND METHODS

### 2.1 Study subjects

This study involved two subject cohorts, namely: a first study cohort recruited in University Hospital of Palermo which included 71 patients with biopsy‐proven NAFLD and 29 controls and a second external cohort recruited in University Hospital of Milan which included 50 NAFLD patients. A liver biopsy was performed to confirm the diagnosis of NAFLD in patients with ultrasound findings of fatty liver and/or persistent (>6 months) elevation of alanine aminotransferase (ALT) or AST. We excluded patients with alcohol‐induced or drug‐induced liver disease autoimmune or viral hepatitis and cholestatic or genetic liver disease from our study. We also excluded patients with current or past consumption of ethanol of more than 20 g per day. This study was conducted according to the Declaration of Helsinki and was approved by the ethics committee of the University Hospital of Palermo and Milan. Before the procedures, a written informed consent was obtained from all patients participating in this study. Further information regarding histopathological evaluation and sample processing can be found as *Supplementary Data*.

## 3 RNA EXTRACTION

We performed RNA extraction from serum samples of study cohorts, from liver biopsies of 12 subjects (four CTRL, four mild NAFLD NAS score ≤ 4 Fibrosis stage = 0, four severe NAFLD NAS ≥ 5 Fibrosis Stage = 3), and finally from HepG2 cells exposed to lipid treatment and their culture media. Total RNA was extracted by using miRNeasy mini kit (Qiagen). Further information regarding RNA extraction protocol can be found as *Supplementary Data*.

## 4 MICROARRAY ANALYSIS

High‐throughput profiling of serum coding/non‐coding RNA, through microarray technology by Clariom D Pico Assay (Thermo Fisher Scientific), was performed on 12 study subjects: four mild NAFLD (NAS ≤ 4, Fibrosis stage = 0), four severe NAFLD (NAS ≥ 5, Fibrosis Stage = 3) and four CTRL. Further information regarding microarray experiments can be found as *Supplementary Data*.

## 5 COMPUTATIONAL ANALYSIS

To understand the function of statistically significant deregulated transcripts for each comparison (mild NAFLD vs CTRL, severe NAFLD vs CTRL, severe vs mild NAFLD), we performed pathway enrichment analysis through Transcriptome Analysis Console v. 4 which retrieves canonical biological pathways from WikiPathways database and establishes *P*‐values using two‐sided Fisher's Exact Test (*P* < 0.05).

## 6 IN VITRO CELL CULTURE EXPERIMENTS

Human hepatoblastoma cell line HepG2 was cultured as previously reported.^27^ To obtain in vitro models of NAFL or NASH, HepG2 at 75% confluence were exposed to a mixture of oleate/palmitate or only palmitate at a final fatty acid concentration of 0.5 mM for 48 h respectively.^27^ ^28^

## 7 SINGLE REAL‐TIME PCR ASSAYS

Validation of candidate coding/non‐coding RNAs both in an independent internal cohort of 88 study subjects (63 NAFLD and 25 CTRL) and an in external cohort of 50 NAFLD patients was performed through real‐time PCR assays (Power SYBR Green RNA‐to‐CT1‐Step Kit Thermo Fisher Scientific) according to the manufacturer's protocol. Candidate transcripts were selected according to the following three criteria: (i) high levels of fluorescence microarray signals; (ii) low *P*‐value in severe NAFLD vs mild NAFLD comparison (*P* ≤ 0.02); (iii) involvement in oxidative stress, inflammatory, apoptotic, authophagy or fibrogenic pathways according to the literature data.^29^, ^30^, ^31^, ^32^, ^33^, ^34^ Validated transcript expression was also analysed, through real‐time PCR assays, in liver biopsies and in NAFL/NASH in vitro models at intracellular and extracellular level.

### 7.1 STATISTICAL ANALYSIS

Differentially expressed (DE) coding and non‐coding RNA identification was performed by Transcriptome Analysis Console (TAC) Software v.4, according to the following parameters: Analysis Type: Expression Gene; Summarization Method: Gene Level – RMA; Gene‐Level *P* < 0.05; ANOVA Method: ebayes. GraphPad Prism (version 6.02) was used to: (i) perform unpaired t test among ‐ΔCt values obtained after single real‐time PCR assays; (ii) perform linear regression analysis between clinical/histological data and candidate coding/non‐coding RNA ‐ΔCt values. Receiver operating characteristics (ROC) curve analysis and multivariate logistic analyses were performed through SPSS PASW Statistics.

## 8 RESULTS

### 8.1 WHOLE TRANSCRIPTOME ANALYSIS

**Figure 1:**
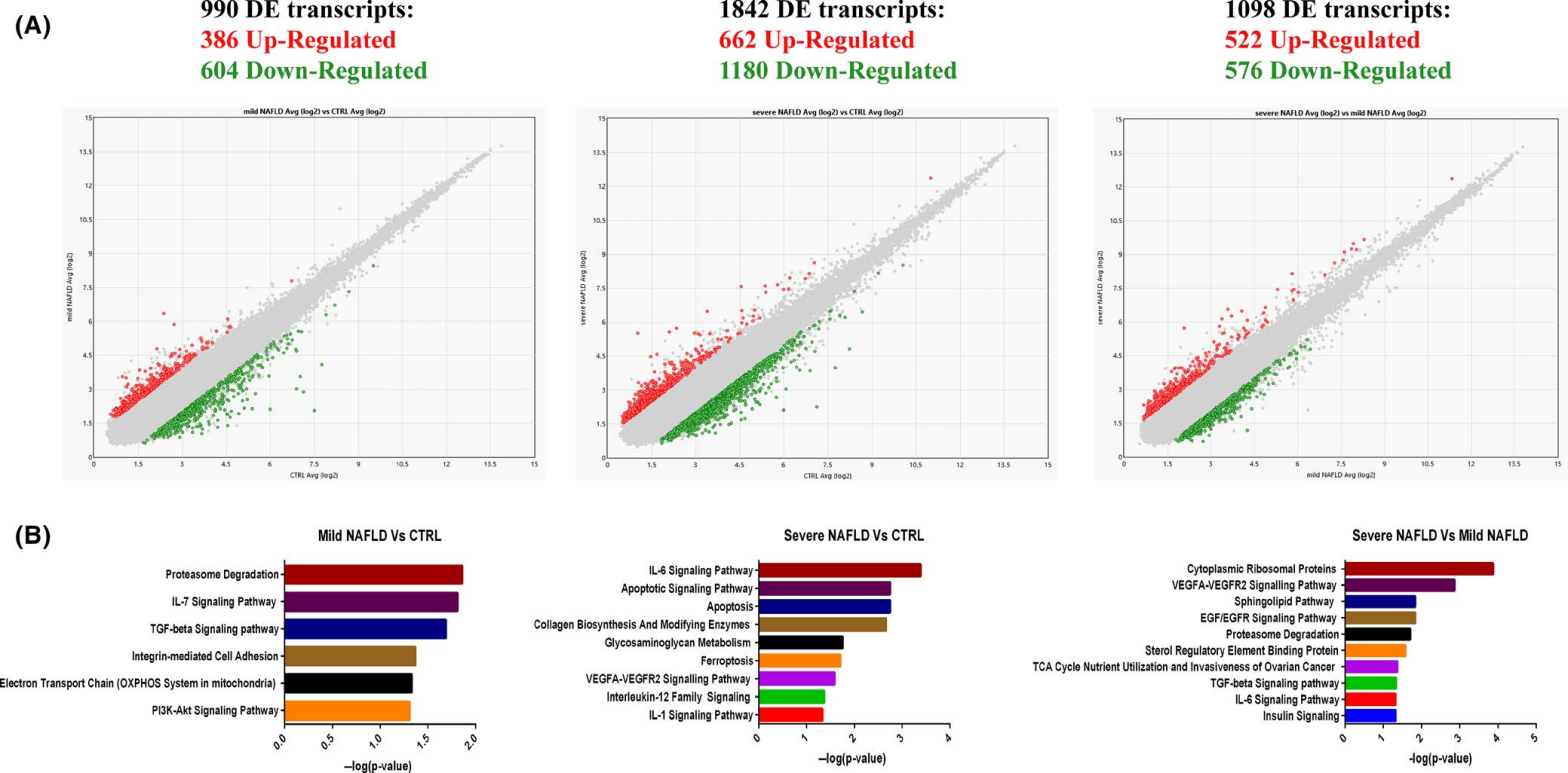
Scatter plots were used to assess the variation between mild NAFLD vs control (left panel); severe NAFLD vs CTRL (middle panel); severe NAFLD vs mild NAFLD (right panel) subjects. The values plotted on the X and Y axes are the averaged normalized signal values in each group (log2 scaled). Red points in the plot indicate > 2.0‑fold up‐regulation of expression, green points indicate > 2.0‑fold down‐regulation of expression and grey points indicate < 2.0‑fold change in expression (panel A). Enriched Pathway analysis. Statistically significant pathways regulated by DE transcripts in mild NAFLD vs CTRL comparison (left panel); severe NAFLD vs CTRL comparison (middle panel); in severe NAFLD vs mild NAFLD comparison (right panel). Values plotted in X axes represent ‐log10 (*P*‐value). Fisher's Exact Test *P* < 0.05 (panel B)

In order to identify novel potential biomarker signatures associated with NAFLD *spectrum*, we performed a whole transcriptome analysis in sera from four mild NAFLD patients (NAS ≤ 4; *F* = 0), four severe NAFLD patients (NAS ≥ 5; *F* = 3) and four healthy matched controls. Statistically significant deregulated transcripts in each comparison are represented as scatter plots in Figure 1A. We identified: 990 deregulated transcripts in mild NAFLD vs CTRL comparison; 1842 deregulated transcripts in severe NAFLD vs CTRL comparison and 1098 deregulated transcripts in severe NAFLD vs mild NAFLD comparison. Fold change (FC) deregulation counts of statistically significant DE coding and non‐coding RNAs (ANOVA Method: ebayes *P* < 0.05) for each comparison are reported in Figure S1. RNA class percentage variations for each comparison are reported in Figure S2. Hierarchical clustering analysis of statistically significant deregulated transcripts showed distinguishable signatures in each comparison: mild NAFLD vs CTRL, severe NAFLD vs CTRL, severe NAFLD vs mild NAFLD (Figures S3, S4 and S5).

## 9 PATHWAY ANALYSIS OF DIFFERENTIALLY EXPRESSED TRANSCRIPTS

To explore the potential functions of deregulated transcripts, we identified significantly enriched biological pathways through Transcriptome Analysis Console. This bioinformatics analysis demonstrated that DE transcripts were statistically significant (Fisher's Exact Test *P* < 0.05) associated with several signalling pathways known to be involved in NASH/Fibrosis pathogenesis including inflammatory pathways (eg interleukins signalling pathways), metabolism deregulation (eg electron transport chain, TCA cycle nutrient utilization, Sterol Responsive Element Binding Protein, insulin signalling), cell death (eg apoptosis, ferroptosis), UPR stress (eg proteasome degradation, cytoplasmic ribosomal proteins), and extracellular matrix biosynthesis/fibrosis (eg collagen biosynthesis and modifying enzymes, VEGFA‐VEGFR signalling pathway, EGF/EGFR signalling pathway) (Figure 1B).

## 10 VALIDATION BY qPCRs

In order to confirm microarray data, we analysed the expression of candidate transcripts through qPCRs in a larger internal independent cohort of 88 subjects (CTRL = 25 and NAFLD = 63) and in an external cohort of 50 NAFLD patients. Clinical and demographic characteristics of each cohort subjects are reported in Table 1. We selected the following transcripts: HBA2, UBE2V1, BNIP3L coding RNAs, RP11‐128N14.5 lncRNA and TGFB2/TGFB2‐OT1 coding/non‐coding RNA. We chose these transcripts because: (i) they presented high levels of fluorescence microarray signals; (ii) they presented a low *P*‐value in severe NAFLD vs mild NAFLD comparison (*P* ≤ 0.02); (iii) they were potentially linked to oxidative stress, inflammatory, apoptotic, authophagy or fibrogenic pathways according to literature data.^29^, ^30^, ^35^ We also selected one ncRNA GenBank ID: AC020558.4 (‘AceView’ database gene name: ‘sherveebu’) as a down‐regulated control molecule.

**Table 1:** Clinical and demographic data of internal and external cohort

		Unaffected controls (25)		NAFLD patients (63)		External cohort NAFLD (50)		*P* value Controls vs NAFLD patients		*P* value NAFLD patients vs external cohort	
Clinical variables											
Age (years)		48.00 ± 10.01		50.65 ± 12.35		52.76 ± 10.00		0.252		0.358	
Gender (% of male)		32%		59%		70%		**0.043**		0.298	
BMI (kg/m^2^)		24.14 ± 2.14		29.87 ± 4.57		29.08 ± 3.83		**0.00000011**		0.378	
AST (IU/I)		20.63 ± 5.81		46.50 ± 27.77		41.10 ± 29.03		**0.00002**		0.348	
ALT (IU/I)		20.71 ± 7.21		78.03 ± 67.09		57.44 ± 46.45		**0.0001**		0.091	
Platelets (×10^9^/I)		248.50 ± 36.96		236.18 ± 87.44		202 ± 72.38		0.234		0.063	
Total cholesterol (mg/dL)		200.94 ± 39.90		191.42 ± 39.74		186.39 ± 41.58		0.387		0.556	
HDL cholesterol (mg/dL)		61.50 ± 10.17		48.54 ± 16.44		46.18 ± 14.31		**0.035**		0.460	
Triglycerides (mg/dL)		93.88 ± 30.27		136.93 ± 74.29		154.90 ± 70.63		**0.023**		0.231	
FIB‐4 score				1.52 ± 1.36		1.61 ± 0.96				0.756	
APRI score				0.38 ± 0.35		0.59 ± 0.58				**0.036**	
LSM – KPa				12.27 ± 11.90							
Histology										
Kleiner steatosis grade (0‐3)										
0				‐		1 (2%)				0.352
1				19 (30%)		11 (22%)				
2				24 (38%)		17 (34%)				
3				29 (46%)		21 (42%)				
Kleiner lobular inflammation score (0‐3)										
0				‐‐‐		3 (6%)				0.550
1				37 (59%)		15 (30%)				
2				21(33%)		27 (54%)				
3				5 (8%)		5 (10%)				
Kleiner Ballooning score (0‐2)										
0				18 (29%)		16 (32%)				0.510
1				33 (52%)		27 (54%)				
2				12 (19%)		7 (14%)				
Kleiner NAS (0‐8)										
≤4				38 (60%)		23 (46%)				0.598
≥5				25 (40%)		27 (54%)				
Kleiner fibrosis stage (0‐4)										
0‐2				37 (59%)		26 (52%)				0.275
3‐4				26 (41%)		24 (48%)				

Clinical and demographic indicators were checked for significant differences by *t* test or Mann‐Whitney *U*‐test, respectively for normally distributed or not normally distributed variables. The Chi‐square test was used for categorical variables. The continuous normally distributed variables were represented as mean ± SD categorical and non‐normal variables were summarized as median and percentage.

The bold values are significance.

**Figure 2:**
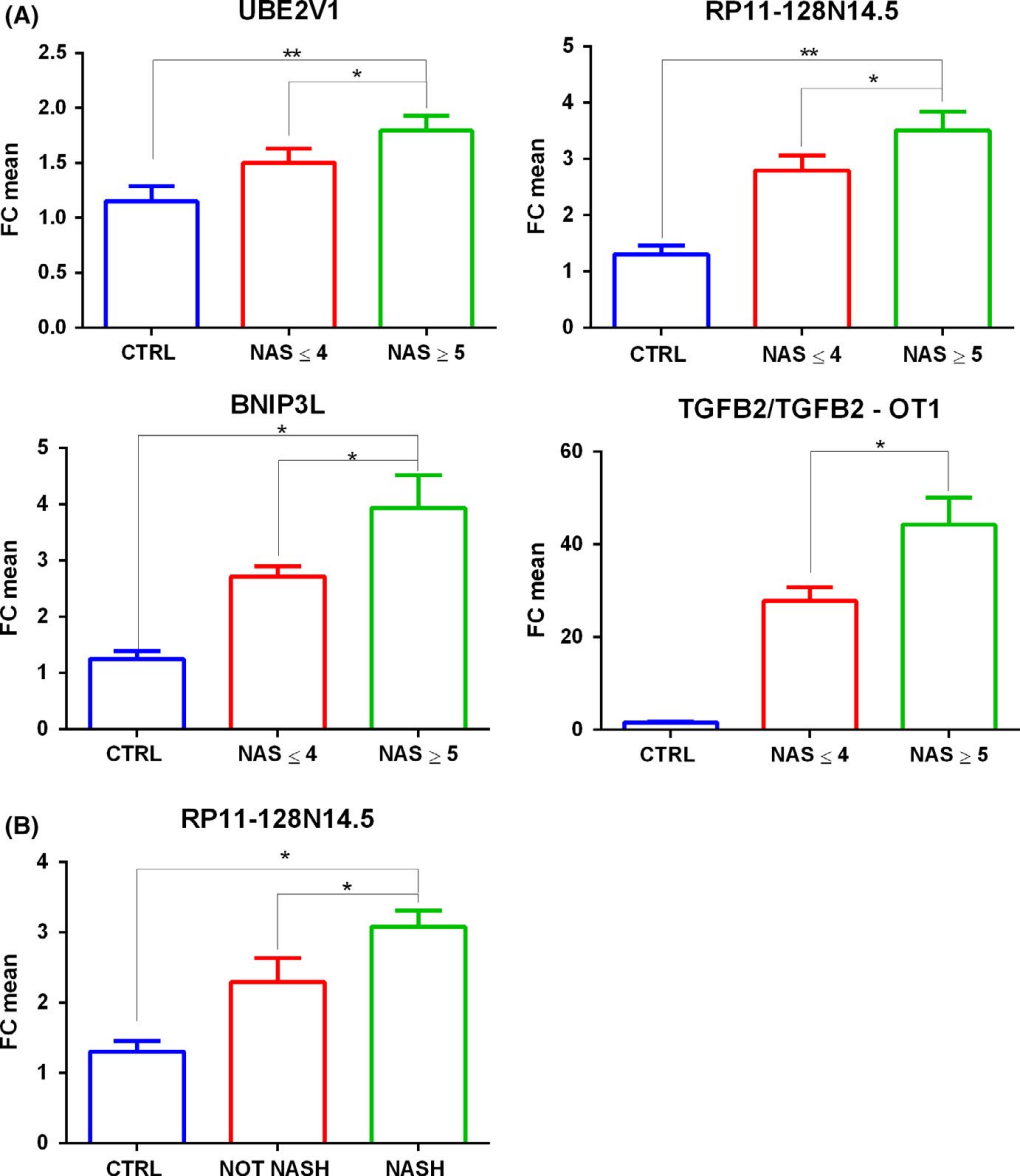
Fold Change of UBE2V1 mRNA, RP11‐128N14.5 lncRNA, BNIP3L mRNA, TGFB2/TGFB2‐OT1 coding/lncRNA validated through real‐time PCRs in serum samples of patients with NAS score ≤ 4 or NAS score ≥ 5 respect to healthy controls (panel A). Fold Change of RP11‐128N14.5 lncRNA validated through real‐time PCRs in serum samples of patients with simple steatosis or defined NASH respect to healthy controls (panel B). *n *= 88:25 CTRL, 38 NAS ≤ 4, 25 NAS ≥ 5.18 NOT NASH, 45 NASH. *t* test *P ≤ 0.05,**P ≤ 0.02

First, we grouped the validation NAFLD cohort according to the presence or absence of NASH (defined as simultaneous presence of steatosis, ballooning and lobular inflammation) and according to Kleiner NAS ≥ 5, associated to defined NASH diagnosis. According to microarray results, we confirmed the up‐regulation of UBE2V1, RP11‐128N14.5, BNIP3L and TGFB2/TGFB2‐OT1 in serum samples of patients with a NAS ≥ 5 with respect to those with NAS ≤ 4 (Figure 2A). Moreover, taking into account NASH diagnosis only RP11‐128N14.5 reached statistical significance (NOT NASH *n* = 18 vs NASH n = 45 FC = 7.13, *P* = 2.00E‐02) (Figure 2B).

**Figure 3:**
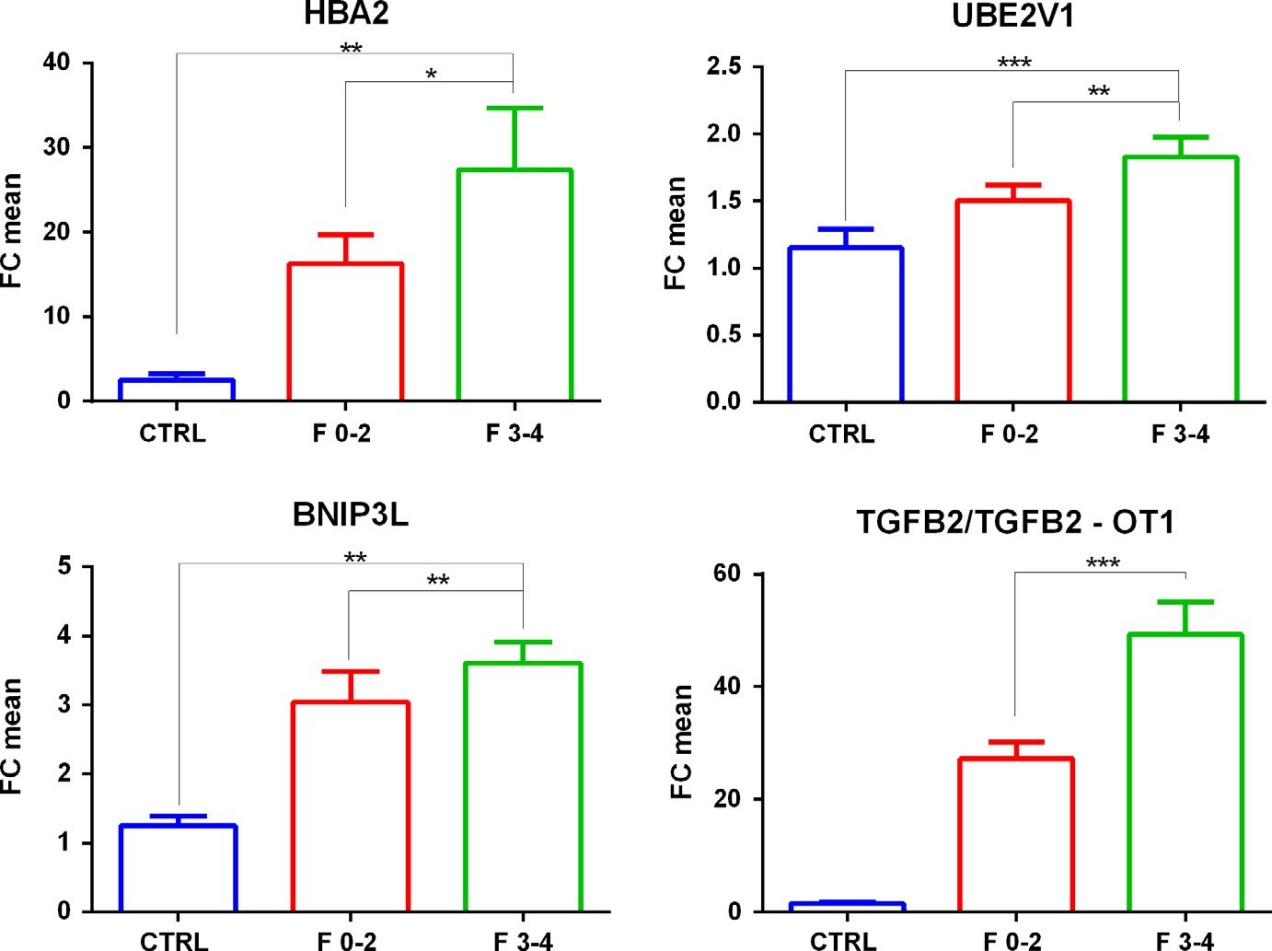
Fold Change of HBA2 mRNA, UBE2V1 mRNA, BNIP3L mRNA, TGFB2/TGFB2‐OT1 coding/ lncRNA validated through real‐time PCRs in serum samples patients with Fibrosis Stages *F* = 0‐2 or *F* = 3‐4 respect to healthy controls. *n *= 88:25 CTRL, 37 *F* = 0‐2, 26 *F* = 3‐4. *t* test *P ≤ 0.05,**P ≤ 0.01,***P ≤ 0.001

We also divided the NAFLD patient cohort according to fibrosis severity. We compared patients with absent or mild to moderate fibrosis (*F* = 0‐2) with respect to those with advanced fibrosis and cirrhosis (*F* = 3‐4). According to this classification, we validated the up‐regulation of HBA2, UBE2V1, BNIP3L and TGFB2/TGFB2‐OT1. Therefore HBA2 reached statistical significance only in *F* = 3‐4 vs *F* = 0‐2 comparison and RP11‐128N14.5 reached statistical significance only in NAS ≥ 5 vs NAS ≤ 4 and NASH vs not NASH comparisons (Figure 3). It is important to note that, analysed transcripts were up‐regulated also in *F* = 3‐4 or NAS ≥ 5 with respect to control comparisons as previously showed in microarray data. Furthermore, we also validated AC020558.4 down‐regulation in NAFLD patients with respect to controls. FC values and *P*‐values of selected coding/non coding RNAs, obtained through microarray analysis and qPCRs, are reported in Table S1.

The up‐regulation of UBE2V1, RP11‐128N14.5, BNIP3L and TGFB2/TGFB2‐OT1 in NAS ≥ 5 vs NAS ≤ 4 patients, RP11‐128N14.5 in NASH vs simple steatosis and the up‐regulation of HBA2, and TGFB2/TGFB2‐OT1 in *F* = 3‐4 vs *F* = 0‐2 patients were also confirmed in the external validation cohort. Transcript FC deregulation and relative *P*‐values obtained in the external validation cohort are reported in Figures S6 and S7.

## 11 VALIDATED TRANSCRIPTS ARE ASSOCIATED WITH CLINICAL MARKERS AND HISTOLOGICAL SCORES

We analysed whether ‐ΔCt values of validated transcripts were associated through a linear regression relationship both with the metabolic syndrome and liver damage‐associated routine biochemical markers as with histological markers used in clinical practice to diagnose and stage NAFLD and fibrosis severity (Table S2).

UBE2V1 expression levels correlated with AST, ALT, triglycerides, Kleiner ballooning and NAS scores (Figure S8). BNIP3L expression was directly associated with Kleiner lobular inflammation, ballooning and fibrosis scores (Figure S9). RP11‐128N14.5 expression was associated with AST, Kleiner ballooning, NAS and fibrosis scores (Figure S10). TGFB2/TGFB2‐OT1 was associated through a positive linear regression relationship with FIB‐4, Liver Stiffness Measurements, Kleiner lobular inflammation and fibrosis scores (Figure S11). Finally, we found an inverse linear relationship among *sherveebu* AC020558.4 expression levels and triglycerides (Figure S12) and between HBA2 and total cholesterol levels. *P*‐values and *R* ^2^ values are reported in Table S2.

## 12 EXPRESSION ANALYSIS OF LIVER TISSUES AND IN VITRO MODELS

In order to further investigate the possible link between validated transcripts and cellular/molecular mechanisms associated with disease evolution, we evaluated whether RNA deregulation observed in serum of NAFLD patients was also present in liver tissue of NAFLD patients and in in vitro models of NAFL and NASH, both at intracellular and extracellular levels. Total RNA was extracted from liver biopsies of 12 subjects (four CTRL, four mild NAFLD NAS ≤ 4 Fibrosis stage = 0 and four severe NAFLD NAS ≥ 5 Fibrosis Stage = 3). We used an in vitro model that we already used in a previous work.^27^ HepG2 cells were exposed for 48 h to a mixture of oleate:palmitate (OA:PA) or only to palmitate (PA), to simulate simple steatosis or NASH respectively.^27^

**Figure 4:**
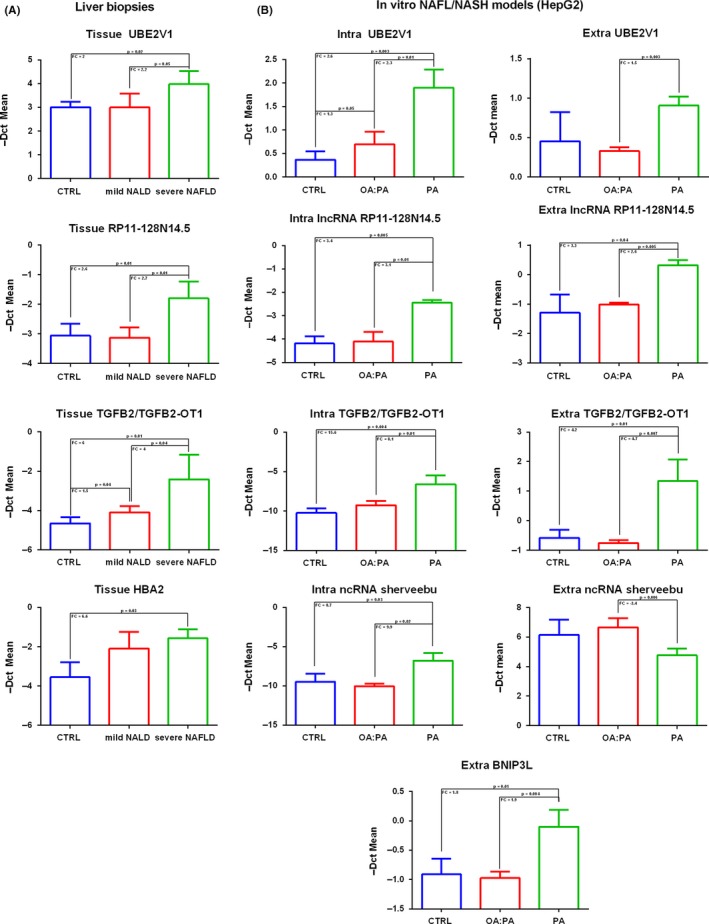
Histograms of coding/noncoding RNA expression levels observed in liver biopsies (*n* = 12, four CTRL, four mild NAFLD: NAS ≤ 4 *F* = 0, four severe NAFLD NAS SCORE ≥ 5 *F* = 3) (panel A) and in OA:PA and PA treated HepG2 with respect to controls at intracellular and extracellular levels (panel B). OA:PA = oleate:palmitate; PA = palmitate; CTRL = control. FC = fold change

As shown in Figure 4A in liver tissue, we observed a statistically significant up‐regulation of UBE2V1 mRNA, RP11‐128N14.5 lncRNA and TGFB2/TGFB2‐OT1 coding/non‐coding RNA in severe NAFLD patients with respect to mild NAFLD patients and controls. HBA2 resulted to be statistically significant up‐regulated only in severe NAFLD vs CTRL comparison. BNIP3L and *sherveebu* AC020558.4 ncRNA did not reach statistical significance in analysed liver biopsies. In accordance with liver tissue data, UBE2V1 mRNA, RP11‐128N14.5 lncRNA and TGFB2/TGFB2‐OT1 coding/non‐coding RNA were up‐regulated in cells treated with PA with respect to those treated with OA:PA and to controls (Figure 4B). Differently from tissue data, intracellular HBA2 expression levels were unaffected by free fatty acid treatments and *sherveebu* AC020558.4 ncRNA presented a statistical significant increasing trend of expression ranging from NAFL to NASH in vitro models.

At the extracellular level, UBE2V1 mRNA and *sherveebu* AC020558.4 ncRNA were statistically significantly deregulated only in PA vs OA:PA condition comparison; medium‐secreted RP11‐128N14.5 lncRNA, BNIP3L mRNA and TGFB2/TGFB2‐OT1 coding/non‐coding RNA were up‐regulated in a statistically significant manner both in PA vs CTRL comparison as in PA vs OA:PA comparison (Figure 4B**)**. Furthermore, it is important to note that HBA2 was undetectable in HepG2 culture medium.

## 13 RECEIVER OPERATING CHARACTERISTICS CURVE ANALYSIS

By performing ROC curve analysis, we investigated, the diagnostic performance of validated transcripts for *F* = 3‐4 or NAS ≥ 5 patient identification in NAFLD validation cohorts. The transcripts that reached statistical significance in ROC curve analysis were: (I)TGFB2/TGFB2‐OT1 (internal cohort: AUC = 0.797, 95% CI = 0.675‐0.918, sensitivity = 65%, specificity = 81.3%, *P* = 3.5E‐04; external cohort: AUC = 0.786, 95% CI = 0.623‐0.950, sensitivity = 62.5%, specificity = 94.4%, *P* = 4.4E‐03) in *F* = 3‐4 vs F = 0‐2 comparison; (II) RP11‐128N14.5 (internal cohort: AUC = 0.706, 95% CI = 0.554‐0.857, sensitivity = 73.7%, specificity = 70.4%, *P* = 1.9E‐02; external cohort: AUC = 0.694, 95% CI = 0.545‐0.843, sensitivity = 78.3%, specificity = 63%, *P* = 2.0E02) and BNIP3L (internal cohort: AUC = 0.676, 95% CI = 0.512‐0.841, sensitivity = 57.9%, specificity = 81.5%, *P* = 4.3E‐02; external cohort: AUC = 0.686, 95% CI = 0.537‐0.835, sensitivity = 51.9%, specificity = 91.3%, *P* = 3.0E02) in NAS ≥ 5 vs NAS ≤ 4 comparison.

**Figure 5:**
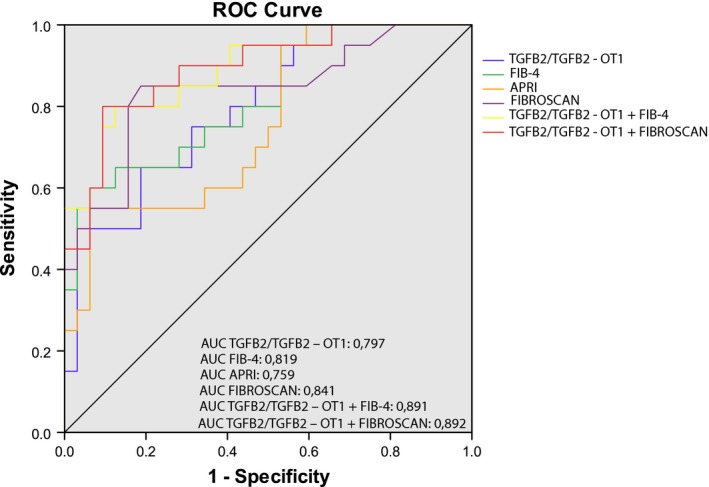
Univariate and Multivariate ROC curve analysis for predicting APRI, FIB‐4, LSM, TGFB2/TGFB2‐OT1, TGFB2/TGFB2‐OT1 + FIB‐4, TGFB2 + Fibroscan as *F* = 3‐4 patient diagnosis biomarkers with respect to *F* = 0‐2 patients. APRI AUC = 0.759 (95% CI = 0.626‐0.893 sensitivity = 55%, specificity = 93.8%, *P* = 1.80E‐03); FIB‐4 AUC = 0.819 (95% CI = 0.700‐0.937 sensitivity = 60%, specificity = 93.8%, *P* = 1.30E‐04); TGFB2/TFB2‐OT1 AUC = 0.797 (95%, CI = 0.675‐0.918 sensitivity = 65%, specificity = 81.3%, *P* = 3.50E‐04); TGFB2/TGFB2‐OT1 + FIB4 AUC = 0.891 (95% CI = 0.799‐0.982, sensitivity = 80%, specificity = 87.5%, *P* = 3.00E‐06). TGFB2/TFB2‐OT1 + Fibroscan AUC = 0.892 (95% CI = 0.802‐0.983 sensitivity = 80%, specificity = 90.6%, *P* = 2.00E‐06)

It is important to note that TGFB2/TGFB2‐OT1 expression levels were associated with the blood‐based index FIB‐4 and LSM used in clinical practice for non‐invasive diagnosis of *F* = 3‐4 stages and histological Kleiner fibrosis stage. Comparison of the AUCs revealed the combinations TGFB2/TGFB2‐OT1 plus FIB‐4 (internal cohort: AUC = 0.891, 95% CI = 0.799‐0.982, sensitivity = 80%, specificity = 87.5%, *P* = 3.0E‐06; external cohort: AUC = 0.889, 95% CI = 0.771‐0.998, sensitivity = 87.5%, specificity = 83.3%, *P* = 1.1E‐04) or TGFB2/TGFB2‐OT1 plus LSM (AUC = 0.892, 95% CI = 0.802‐0.983, sensitivity = 80%, specificity = 90.6%, *P* = 2.0E‐06) were superior to APRI (internal cohort: AUC = 0.759, 95% CI = 0.626‐0.893, sensitivity = 55%, specificity = 93.8%, *P* = 1.8E‐0.3; external cohort: AUC = 0.667, 95% CI = 0.479‐0.854, sensitivity = 68.8%, specificity = 66.7%, *P* = 9.8E‐02); FIB‐4 (internal cohort: AUC = 0.819, 95% CI = 0.700‐0.937, sensitivity = 60%, specificity = 93.8%, *P* = 1.3E‐0.4; external cohort: AUC = 0.839, 95% CI = 0.698‐0.979, sensitivity = 93.8%, specificity = 66.7%, *P* = 7.7E‐04); TGB2/TGFB2‐OT1 (internal cohort: AUC = 0.797, 95% CI = 0.675‐0.918, sensitivity = 65%, specificity = 81.3%, *P* = 3.5E‐04; external cohort: AUC = 0.786, 95% CI = 0.623‐0.950, sensitivity = 62.5%, specificity = 94.4%, *P* = 4.4E‐03) and LSM (AUC = 0.841, 95% CI = 0.723 −0.960, sensitivity = 85%, specificity = 81.3%, *P* = 4.0E‐05) (Figure 5, Figure S13).

In NAS ≥ 5 patient discrimination, RP11‐128N14.5 diagnostic performance was comparable or superior to AST performance (internal cohort: AUC = 0.707, 95% CI = 0.554‐0.860 sensitivity = 73.7%, specificity = 74.1%, *P* = 1.8E‐02; external cohort: AUC = 0.659, 95% CI = 0.470‐0.849, sensitivity = 38.9, specificity = 99%, *P* = 8.3E02) and superior to ALT (internal cohort: AUC = 0.367, 95% CI = 0.203‐0.532, sensitivity = 94.7%, specificity = 3.7%, *P* = 1.3E‐01; external cohort: AUC = 0.633, 95% CI = 0.449‐0.817, sensitivity = 55.6%, specificity = 82.6%, *P* = 1.5E01). Combination of RP11‐128N14.5 with AST or ALT did not improve its diagnostic performance.

In NASH vs not NASH comparison, RP11‐128N14.5 did not reach statistical significance; however, its AUC (internal cohort: AUC = 0.632, 95% CI = 0.477‐0.787, sensitivity = 86.7%, specificity = 38.9%, *P* = 1.0E‐01; external cohort: AUC = 0.650, 95% CI = 0.480‐0.819, sensitivity = 53.3%, specificity = 4.3%, *P* = 9.7E‐02) was superior with respect to ALT (internal cohort: AUC = 0.412, 95% CI = 0.258‐0.567, sensitivity = 25%, specificity = 83.3%. *P* = 2.9E‐01, external cohort: AUC = 0.567, 95% CI = 0.390‐0.744, sensitivity = 57.7%, specificity=73.3%, *P* = 4.8E‐01) and similar to AST (AUC = 0.558, 95% CI = 0.397‐0.720, sensitivity = 69.2%, specificity = 55.6%, *P* = 4.8E‐01; external cohort AUC = 0.672, 95% CI = 0.503‐0.842, sensitivity = 62.5%, specificity = 80%; *P* = 7.3E‐02). Combination of RP11‐128N14.5 with AST or ALT in both cohorts decreased its AUC: 0.5 *P* = 1.

## 14 DISCUSSION

In this multiphase case‐control study, we performed an initial screening of coding mRNAs and ncRNAs in biopsy‐proven NAFLD patients at different stages of histopathological severity and controls (n = 12: 4 CTRL, 4 NAS ≤ 4 *F* = 0, 4 NAS ≥ 5 *F* = 3). Interestingly, performing computational analysis, we observed that deregulated transcripts, resulting from this initial screening, resulted to be statistically associated to key pathways known to be involved in fibrosis and NASH physiopathogenesis such as inflammation, UPR stress and metabolism. To better characterize our results, we chose, for subsequent qPCR validation, five DE transcripts in severe NAFLD vs mild NAFLD with a very compelling *P*‐value (≤ 0.02) and potentially linked to NASH progression and fibrogenic pathways. The potential of validated transcripts for predicting disease severity (NAS ≥ 5) or advanced fibrosis (*F* = 3‐4) in NAFLD patients was also evaluated. Interestingly, combinations of TGFB2/TGFB2‐OT1 plus FIB‐4 (AUC = 0.891, *P* = 3.00E‐06) or TGFB2/TGFB2‐OT1 plus LSM (AUC = 0.892, *P* = 2.00E‐06) were able to powerfully discriminate patients with F 3‐4 with respect to patients F 0‐2. Therefore, the combination of coding/non‐coding RNA expression values and routine clinical data could be conveniently applied as a diagnostic tool for non‐invasive screening of NAFLD patients according to fibrosis stage. Moreover, the best‐performing transcript for NAS ≥ 5 patient identification, RP11‐128N14.5, achieved an AUC similar to AST, and their combined use did not improve the diagnostic power. Therefore, the identification of novel NASH diagnosis biomarkers is still open and needs to be addressed.

In order to determine a possible link between selected coding/non‐coding RNAs and synergic and complex mechanisms underlying disease and fibrosis progression, we tested the hypothesis of a statistical association between validated transcript expression and histological features of NAFLD *spectrum*. In our study, we observed that UBE2V1, BNIP3L, RP11‐128N14.5, TGFB2/TGFB2‐OT1 were correlated with Kleiner histological scores: Kleiner NAS correlated with UBE2V1 (*P* = 4.0E‐03) and RP11‐128N14.5 (*P* = 3.10E‐02); Kleiner lobular inflammation score correlated with BNIP3L (*P* = 8.00E‐03) and TGFB2/TGFB2‐OT1 (*P* = 3.10E‐02); Kleiner ballooning score correlated with UBE2V1 (*P* = 4.50E‐02), BNIP3L (*P* = 2.60E‐02), RP11‐128N14.5 (*P* = 1.20E‐02); Kleiner fibrosis score correlated with BNIP3L (*P* = 3.60E‐02), RP11‐128N14.5 (*P* = 3.70E‐02) and TGFB2/TGFB2‐OT1 (*P* = 1.60E‐02). The statistical correlation with histological scores of disease severity and fibrosis stages suggests that analysed transcripts could be potentially associated with NASH and hepatic fibrogenesis molecular features.

Explaining the relationship between RNA extracellular expression and affected tissues is a difficult challenge. We tried to respond to this question by verifying whether RNA deregulation in patients could be somehow mirrored in liver tissue of NAFLD subjects and into in vitro models of NAFLD disease, both at intracellular and extracellular levels.

María José Gómez‐Lechón *et al*,^36^ and also our research group,^27^ validated differential in vitro models of NAFL and NASH. More in detail, we treated HepG2 cells with an oleate/palmitate mixture or only palmitate in order to mimic NAFL or NASH respectively.

Interestingly, switching from control conditions to OA:PA and PA‐treated cells, the RNAs secreted into the medium partially reflected the expression trends observed in serum samples from healthy controls and mild and severe NAFLD patients. In our in vitro model, we also found that the cellular expression of mRNA UBE2V1, lncRNA RP11‐128N14.5, ncRNA *sherveebu* AC020558.4, TGFB2/TGFB2‐OT1 coding/non‐coding RNA progressively increased, switching from CTRL to OA:PA and PA conditions. Saturated free fatty acid palmitate, which is mainly responsible for lipotoxic effects compared to unsaturated fatty acid oleate, seems to trigger an induction of UBE2V1 mRNA, RP11‐128N14.5 lncRNA and TGFB2/TGFB2‐OT1 expression. Deregulation of previous validated coding/non‐coding RNA in our in vitro models, further supports the statistically significant association between analysed transcripts in patient sera and clinical histological scores of disease severity, and paves the way to explore the potential involvement of analysed RNA molecules in molecular pathways of disease evolution through transient or permanent inhibition methodology, especially for ncRNAs, whose function and involvement in NAFLD *spectrum* have not yet been characterized.

NASH affects not only hepatocytes in the liver, but it involves several other hepatic cell types (eg Hepatic Stellate Cells, innate immune liver cells: Kupffer cells, natural killer cells) and other tissues/organs (eg gut, adipose tissue, circulating inflammatory cells); therefore analysed transcript deregulation in cellular models of NAFL/NASH represents only an attempt to explain the increased serum lncRNA/mRNA levels which could arise from others hepatic cells, extra‐hepatic tissues, or as a result of cumulative effects. To get further insight on the potential liver origin of deregulated transcripts and the relationship among them and biological/molecular mechanisms underlying NASH/Fibrosis severity, we analysed transcript expression in liver biopsies of NAFLD patients and healthy controls. Interestingly UBE2V1, RP11‐128N14.5, TGFB2/TGFB2‐OT1 were up‐regulated in severe NAFLD patient with respect to mild NAFLD and controls. HBA2 was up‐regulated only in severe NAFLD patient vs controls.

A link between haemoglobin levels and NASH already exists. Akyuz *et al* reported that increased serum haemoglobin is the only independent predictor of both NASH and hepatic fibrosis in NAFLD patients with BMI < 25 kg/m^2^.^37^ Trak‐Smayra and colleagues performed a proteomic study in a cohort of obese patients in the presence or absence of liver lesions: they observed an increase of free haemoglobin subunits ranging from obese patients without liver lesion to those with simple steatosis and then to NASH, which returned to normal values after weight loss.^38^ Whether increased free serum haemoglobin subunits is owing to the increased hemolysis or increased gene and protein expression occurring in the affected liver, needs to be elucidated. In support of the first hypothesis, it has been reported that oxidative stress induces an increased erythrocyte susceptibility to haemolysis in animal models of NASH 30 and in obese subjects.^39^ In support of the second hypothesis, Liu *et al* reported an increased expression of HBA1 and HBB in liver biopsies of NASH patients with respect to controls. They also demonstrated that H_2_O_2 _treatment of HepG2 cells increases gene and protein expression of HBA1 and HBB. Furthermore, haemoglobin over‐expression induced oxidative stress decrease.^29^ In accordance with these results we observed a statistically significant up‐regulation of HBA2 transcript in severe NAFLD patients respect to controls. However, despite the fact that palmitate is a known oxidative stress inducer,^40^ we did not observe any HBA2 gene expression alteration in our in vitro models and we were not able to detect HBA2 expression in culture medium. Probably, in NAFLD, the main contribution of the increase in HBA2 gene expression is given by other cell types, different from hepatocytes, inside the affected liver tissue.

Although any direct link among UBE2V1 gene expression and NAFLD has not been reported, UBE2V1 is involved in non‐canonical IKK poly‐ubiquitination, which in turn activates NF‐kappa‐B cascade, which is one of the key pro‐inflammatory signalling pathways active in NASH.^41^

There are not any literature data concerning the involvement of RP11‐128N14.5 in NAFLD. RP11‐128N14.5 is a long intronic RNA transcribed in the same direction of its protein‐coding host gene RAP2A. RP11‐128N14.5, as already reported for several lncRNAs, could have a role in the epigenetic regulation of its host gene locus. There are no published data on the association between the RAP2A coding gene and NAFLD *spectrum*; however, it has been reported that in hepatocellular carcinoma, RAP2A regulates MAP4K4, which leads to JNK and NF‐κB signalling activation which are the members of common molecular pathways of NASH pathogenesis.

TGF‐β is a homodimer that exists in three different isoforms (‐β1, ‐β2 and ‐β3) in mammals and all TGF‐β ligands act through the same receptor signalling systems. It has been widely demonstrated that, in the liver, TGF‐β signalling is involved in the fibrogenic response through hepatic stellate cell activation. Furthermore, it has been reported that TGF‐β signalling in hepatocytes contributes to hepatocyte death and lipid accumulation that promote the development of NASH.^42^ In real‐time validation experiments, we used primers able to amplify both mRNA encoding for TGFB2 and lncRNA TGFB2‐Overlapping Transcript 1, a newly discovered lncRNA deriving from the 3'‐UTR of TGFB2. We found that levels of TGFB2/TGFB2‐OT1 were highly increased in serum samples of patients with a NAS ≥ 5 and fibrosis *F* = 3‐4.

Several studies have highlighted the role of TGFB2‐OT1 in the regulation of autophagy, apoptotic, inflammatory and fibrogenic pathways, all known to be involved in NAFLD progression.^43^ It has been reported that TGFB2‐OT1 is involved in the negative regulation of apoptosis and positive regulation of autophagy and inflammation in vascular endothelial cells.^31^, ^44^ Long Xiang Tu and colleagues reported the up‐regulation of TGFB2‐OT1 in fibroblasts derived from hypertrophic scars, suggesting its involvement in excessive proliferation of fibroblasts and accumulation of extracellular matrix.^45^ As concerns TGFB2, its pro‐fibrogenic role and/or its increased gene or protein expression has been reported in lens fibrosis, kidney fibrosis, cardiac fibrosis and liver fibrosis.^46^

Several studies reported the involvement of BNIP3L in the regulation of pathways associated with NASH pathogenesis, specifically, apoptosis, necrosis, autophagy and fibrosis. Several studies supported BNIP3L involvement in apoptosis and necrosis of cardiomyocyte in heart diseases.^47^ HIF1b‐dependent BNIP3L increased gene and protein expression has been reported in primary cultured hepatocytes exposed to ethanol and in mice models of alcohol‐induced liver injury and steatosis.^48^ Although these data support the potential activation of this pathway in liver steatosis, we did not observe its activation in liver biopsies and in in vitro model at intracellular level. Concerning the pro‐fibrogenic role of BNIP3L, Weili Liu and colleagues reported that the expression of BNIP3L is increased in cardiac fibrosis.^32^

Although the major limitation of our study is the small sample size, our results may contribute to increase the understanding of NAFLD physiopathology. Our qPCR results, or other results deriving from microarray analysis or from similar studies, need to be validated in larger patient cohorts with a clearer stratification according to NAS (NAS ≤ 3 vs NAS ≥ 5). Serum RNAs should be analysed in in vivo animal models in which genetic and environmental confounding effects should be minimized. Finally, intracellular RNAs should be assessed in liver biopsies of larger patient cohort. Notwithstanding these limitations, our data contribute to expand the knowledge on molecular mechanisms associated with NAFLD pathophysiology. Moreover, they could provide the evidence for identification of novel biomarkers as future possible alternatives to liver biopsy.

## CONFLICT OF INTEREST

The authors do not have any disclosures to report.

## References

[1] Younossi Z , Stepanova M , Ong JP , et al. Nonalcoholic steatohepatitis is the fastest growing cause of hepatocellular carcinoma in liver transplant candidates. Clin Gastroenterol Hepatol. 2019;17(4):748–755, e3. 29908364 10.1016/j.cgh.2018.05.057

[2] Tesfay M , Goldkamp WJ , Neuschwander‐Tetri BA . NASH: the emerging most common form of chronic liver disease. Mo Med. 2018;115(3):225–229. 30228727 PMC6140162

[3] Perumpail BJ , Khan MA , Yoo ER , Cholankeril G , Kim D , Ahmed A Clinical epidemiology and disease burden of nonalcoholic fatty liver disease. World J Gastroenterol. 2017;23(47):8263–8276. 29307986 10.3748/wjg.v23.i47.8263 PMC5743497

[4] Wieckowska A , McCullough AJ , Feldstein AE . Noninvasive diagnosis and monitoring of nonalcoholic steatohepatitis: present and future. Hepatology. 2007;46(2):582–589. 17661414 10.1002/hep.21768

[5] Papagianni M , Sofogianni A , Tziomalos K . Non‐invasive methods for the diagnosis of nonalcoholic fatty liver disease. World J Hepatol. 2015;7(4):638–648. 25866601 10.4254/wjh.v7.i4.638 PMC4388992

[6] Cusi K , Chang Z , Harrison S , et al. Limited value of plasma cytokeratin‐18 as a biomarker for NASH and fibrosis in patients with non‐alcoholic fatty liver disease. J Hepatol. 2014;60(1):167–174. 23973932 10.1016/j.jhep.2013.07.042

[7] Maurice J , Manousou P . Non‐alcoholic fatty liver disease. Clin Med. 2018;18(3):245–250. 10.7861/clinmedicine.18-3-245 PMC6334080 29858436

[8] Petta S , Wong V‐S , Cammà C , et al. Improved noninvasive prediction of liver fibrosis by liver stiffness measurement in patients with nonalcoholic fatty liver disease accounting for controlled attenuation parameter values. Hepatology. 2017;65(4):1145–1155. 27639088 10.1002/hep.28843

[9] Hackl M , Heilmeier U , Weilner S , Grillari J . Circulating microRNAs as novel biomarkers for bone diseases ‐ Complex signatures for multifactorial diseases? Mol Cell Endocrinol. 2016;432:83–95. 26525415 10.1016/j.mce.2015.10.015

[10] Shi T , Gao G , Cao Y . Long noncoding RNAs as novel biomarkers have a promising future in cancer diagnostics. Dis Markers. 2016;2016:9085195. 27143813 10.1155/2016/9085195 PMC4842029

[11] Kishikawa T , Otsuka M , Ohno M , Yoshikawa T , Takata A , Koike K . Circulating RNAs as new biomarkers for detecting pancreatic cancer. World J Gastroenterol. 2015;21(28):8527–8540. 26229396 10.3748/wjg.v21.i28.8527 PMC4515835

[12] Tsui NB , Ng EK , Lo YM . Stability of endogenous and added RNA in blood specimens, serum, and plasma. Clin Chem. 2002;48(10):1647–1653. 12324479

[13] Hasselmann DO , Rappl G , Tilgen W , Reinhold U . Extracellular tyrosinase mRNA within apoptotic bodies is protected from degradation in human serum. Clin Chem. 2001;47(8):1488–1489. 11468248

[14] Arita T , Ichikawa D , Konishi H , et al. Circulating long non‐coding RNAs in plasma of patients with gastric cancer. Anticancer Res. 2013;33(8):3185–3193. 23898077

[15] Tong Y‐S , Wang X‐W , Zhou X‐L , et al. Identification of the long non‐coding RNA POU3F3 in plasma as a novel biomarker for diagnosis of esophageal squamous cell carcinoma. Mol Cancer. 2015;14:3. 25608466 10.1186/1476-4598-14-3 PMC4631113

[16] Wetmore BA , Brees DJ , Singh R , et al. Quantitative analyses and transcriptomic profiling of circulating messenger RNAs as biomarkers of rat liver injury. Hepatology. 2010;51(6):2127–2139. 20235334 10.1002/hep.23574

[17] Wang WT , Sun YM , Huang W , He B , Zhao YN , Chen YQ . Genome‐wide long non‐coding RNA analysis identified circulating LncRNAs as novel non‐invasive diagnostic biomarkers for Gynecological disease. Sci Rep. 2016;6:23343. 26987697 10.1038/srep23343 PMC4796908

[18] Chen G , Yu D , Nian X , et al. LncRNA SRA promotes hepatic steatosis through repressing the expression of adipose triglyceride lipase (ATGL). Sci Rep. 2016;6:35531. 27759039 10.1038/srep35531 PMC5069493

[19] Sun C , Liu X , Yi Z , et al. Genome‐wide analysis of long noncoding RNA expression profiles in patients with non‐alcoholic fatty liver disease. IUBMB Life. 2015;67(11):847–852. 26472541 10.1002/iub.1442

[20] Zhang Y , Luo G , Zhang YI , et al. Critical effects of long non‐coding RNA on fibrosis diseases. Exp Mol Med. 2018;50(1):e428. 29350677 10.1038/emm.2017.223 PMC5799794

[21] Yulan Z , SUTHAT LIANGPUNSAKUL, LI WANG . WITHDRAWN: long noncoding RNAs in liver metabolism and liver disease. Current Status. Liver Research. 2017;1(3):163–167. 29576888 10.1016/j.livres.2017.09.001 PMC5863923

[22] Moylan CA , Pang H , Dellinger A , et al. Hepatic gene expression profiles differentiate presymptomatic patients with mild versus severe nonalcoholic fatty liver disease. Hepatology. 2014;59(2):471–482. 23913408 10.1002/hep.26661 PMC3982589

[23] Liu XL , Ming YN , Zhang JY , Chen XY , Zeng MD , Mao YM . Gene‐metabolite network analysis in different nonalcoholic fatty liver disease phenotypes. Exp Mol Med. 2017;49(1):e283. 28082742 10.1038/emm.2016.123 PMC5291835

[24] Arendt BM , Comelli EM , Ma D , et al. Altered hepatic gene expression in nonalcoholic fatty liver disease is associated with lower hepatic n‐3 and n‐6 polyunsaturated fatty acids. Hepatology. 2015;61(5):1565–1578. 25581263 10.1002/hep.27695

[25] Teufel A , Itzel T , Erhart W , et al. Comparison of gene expression patterns between mouse models of Nonalcoholic fatty liver disease and liver tissues from patients. Gastroenterology. 2016;151(3):513–525, e0. 27318147 10.1053/j.gastro.2016.05.051

[26] Wang G , Li B , Hao Y , Zhi J , He C , Xu C . Correlation analysis between gene expression profile of high‐fat emulsion‐induced non‐alcoholic fatty liver and liver regeneration in rat. Cell Biol Int. 2013;37(9):917–928. 23619824 10.1002/cbin.10118

[27] Di Mauro S , Ragusa M , Urbano F , et al. Intracellular and extracellular miRNome deregulation in cellular models of NAFLD or NASH: clinical implications. Nutrition, metabolism, and cardiovascular diseases : NMCD. 2016;26(12):1129–1139. 10.1016/j.numecd.2016.08.004 27756518

[28] Filippello A , Urbano F , Di Mauro S , et al. Chronic exposure to palmitate impairs insulin signaling in an intestinal L‐cell Line: a possible shift from GLP‐1 to glucagon production. Int J Mol Sci. 2018;19(12):3791. 10.3390/ijms19123791 PMC6321596 30487448

[29] Liu W , Baker SS , Baker RD , Nowak NJ , Zhu L . Upregulation of hemoglobin expression by oxidative stress in hepatocytes and its implication in nonalcoholic steatohepatitis. PLoS ONE. 2011;6(9):e24363. 21931690 10.1371/journal.pone.0024363 PMC3171444

[30] Otogawa K , Kinoshita K , Fujii H , et al. Erythrophagocytosis by liver macrophages (Kupffer cells) promotes oxidative stress, inflammation, and fibrosis in a rabbit model of steatohepatitis: implications for the pathogenesis of human nonalcoholic steatohepatitis. Am J Pathol. 2007;170(3):967–980. 17322381 10.2353/ajpath.2007.060441 PMC1864892

[31] Huang ShuYa , Lu W , Ge DI , et al. A new microRNA signal pathway regulated by long noncoding RNA TGFB2‐OT1 in autophagy and inflammation of vascular endothelial cells. Autophagy. 2015;11(12):2172–2183. 26565952 10.1080/15548627.2015.1106663 PMC4835209

[32] Liu W , Wang X , Mei Z , et al. BNIP3L promotes cardiac fibrosis in cardiac fibroblasts through [Ca(2+)]i‐TGF‐beta‐Smad2/3 pathway. Sci Rep. 2017;7(1):1906. 28507335 10.1038/s41598-017-01936-5 PMC5432493

[33] Chinnadurai G , Vijayalingam S , Gibson SB . BNIP3 subfamily BH3‐only proteins: mitochondrial stress sensors in normal and pathological functions. Oncogene. 2008;27(Suppl 1):S114–S127. 19641497 10.1038/onc.2009.49 PMC2925272

[34] Dropmann A , Dediulia T , Breitkopf‐Heinlein K , et al. TGF‐beta1 and TGF‐beta2 abundance in liver diseases of mice and men. Oncotarget. 2016;7(15):19499–19518. 26799667 10.18632/oncotarget.6967 PMC4991397

[35] Huang J , Manning BD . The TSC1‐TSC2 complex: a molecular switchboard controlling cell growth. Biochem J. 2008;412(2):179–190. 18466115 10.1042/BJ20080281 PMC2735030

[36] Gomez‐Lechon MJ , Donato MT , Martinez‐Romero A , Jimenez N , Castell JV , O'Connor JE . A human hepatocellular in vitro model to investigate steatosis. Chem Biol Interact. 2007;165(2):106–116. 17188672 10.1016/j.cbi.2006.11.004

[37] Akyuz U , Yesil A , Yilmaz Y . Characterization of lean patients with nonalcoholic fatty liver disease: potential role of high hemoglobin levels. Scand J Gastroenterol. 2015;50(3):341–346. 25540973 10.3109/00365521.2014.983160

[38] Trak‐Smayra V , Dargere D , Noun R , et al. Serum proteomic profiling of obese patients: correlation with liver pathology and evolution after bariatric surgery. Gut. 2009;58(6):825–832. 18403495 10.1136/gut.2007.140087

[39] Cazzola R , Rondanelli M , Russo‐Volpe S , Ferrari E , Cestaro B . Decreased membrane fluidity and altered susceptibility to peroxidation and lipid composition in overweight and obese female erythrocytes. J Lipid Res. 2004;45(10):1846–1851. 15231850 10.1194/jlr.M300509-JLR200

[40] Urbano F , Bugliani M , Filippello A , et al. Atorvastatin but not pravastatin impairs mitochondrial function in human pancreatic islets and rat beta‐cells. direct effect of oxidative. Stress. Scientific Reports. 2017;7(1):11863. 28928397 10.1038/s41598-017-11070-x PMC5605712

[41] Farrell GC , van Rooyen D , Gan L , Chitturi S . NASH is an inflammatory disorder: pathogenic, prognostic and therapeutic implications. Gut and liver. 2012;6(2):149–171. 22570745 10.5009/gnl.2012.6.2.149 PMC3343154

[42] Yang L , Roh YS , Song J , et al. Transforming growth factor beta signaling in hepatocytes participates in steatohepatitis through regulation of cell death and lipid metabolism in mice. Hepatology. 2014;59(2):483–495. 23996730 10.1002/hep.26698 PMC3946696

[43] Fabregat I , Moreno‐Caceres J , Sanchez A , et al. TGF‐beta signalling and liver disease. FEBS J. 2016;283(12):2219–2232. 26807763 10.1111/febs.13665

[44] Ma H , Su L , Zhang S , Kung H , Miao J . Inhibition of ANXA7 GTPase activity by a small molecule promotes HMBOX1 translation of vascular endothelial cells in vitro and in vivo. Inter J Biochem Cell Biol. 2016;79:33–40. 10.1016/j.biocel.2016.08.010 27506770

[45] Tu L , Huang Q , Fu S , Liu D . Aberrantly expressed long noncoding RNAs in hypertrophic scar fibroblasts in vitro: A microarray study. Int J Mol Med. 2018;41(4):1917–1930. 29393369 10.3892/ijmm.2018.3430 PMC5810216

[46] Pohlers D , Brenmoehl J , Loffler I , et al. TGF‐beta and fibrosis in different organs ‐ molecular pathway imprints. Biochem Biophys Acta. 2009;1792(8):746–756. 19539753 10.1016/j.bbadis.2009.06.004

[47] Diwan A , Wansapura J , Syed FM , et al, 2nd. Nix‐mediated apoptosis links myocardial fibrosis, cardiac remodeling, and hypertrophy decompensation. Circulation. 2008;117(3):396–404. 18178777 10.1161/CIRCULATIONAHA.107.727073 PMC2538800

[48] Ni HM , Bhakta A , Wang S , et al. Role of hypoxia inducing factor‐1beta in alcohol‐induced autophagy, steatosis and liver injury in mice. PLoS ONE. 2014;9(12):e115849. 25536043 10.1371/journal.pone.0115849 PMC4275262

