# Supplementary figures and images for: Serum coding and non‐coding RNAs as biomarkers of NAFLD and fibrosis severity
Source: Liver Int. 2019 Jun 26;39(9):1742–54. doi: 10.1111/liv.14167 (PMC6771597; doi:10.1111/liv.14167)

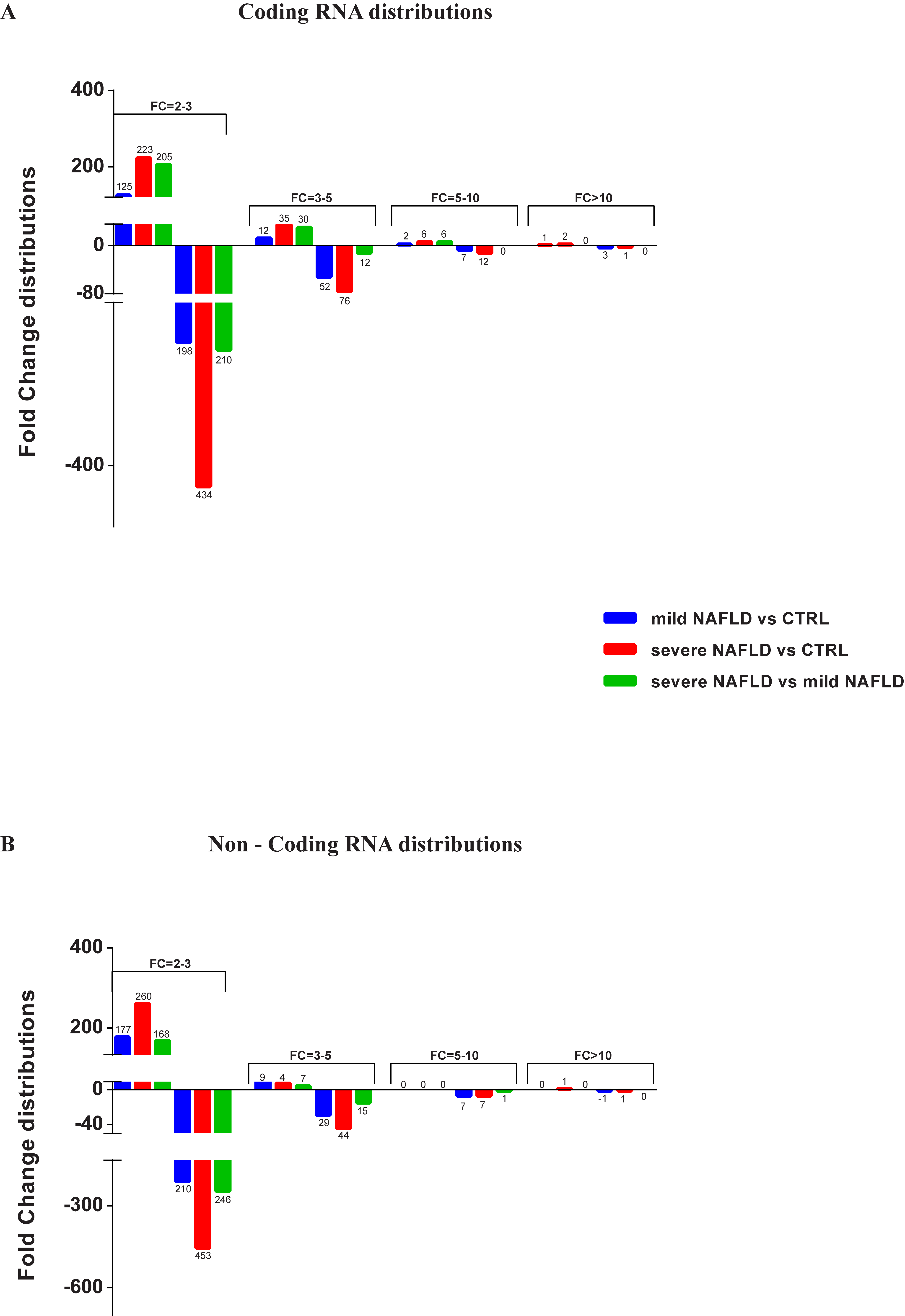

Supplement: Supplementary file 1 [file LIV-39-1742-s001.tif]

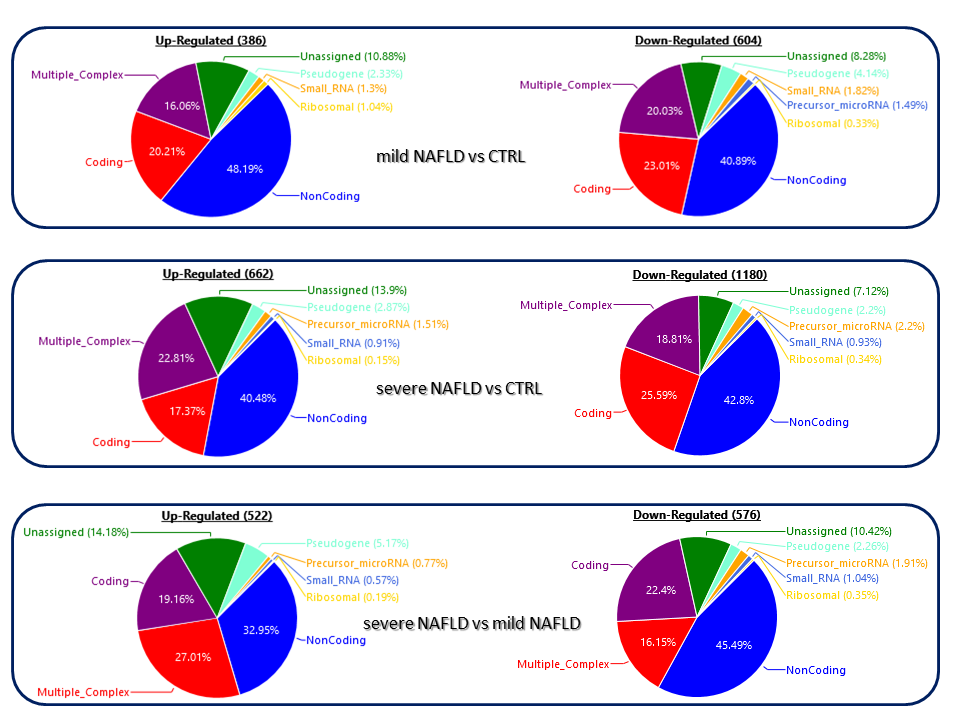

Supplement: Supplementary file 2 [file LIV-39-1742-s002.TIF]

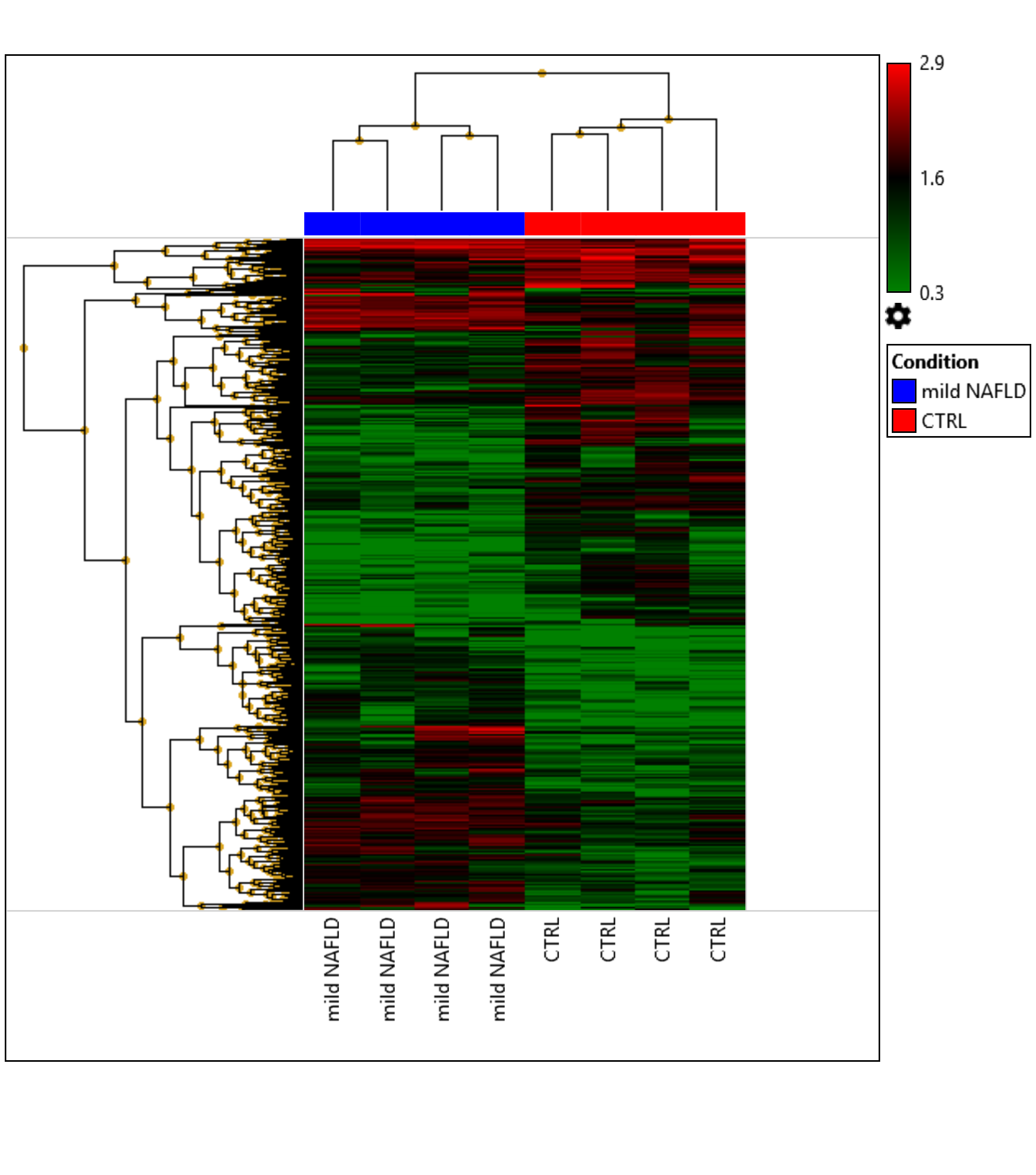

Supplement: Supplementary file 3 [file LIV-39-1742-s003.png]

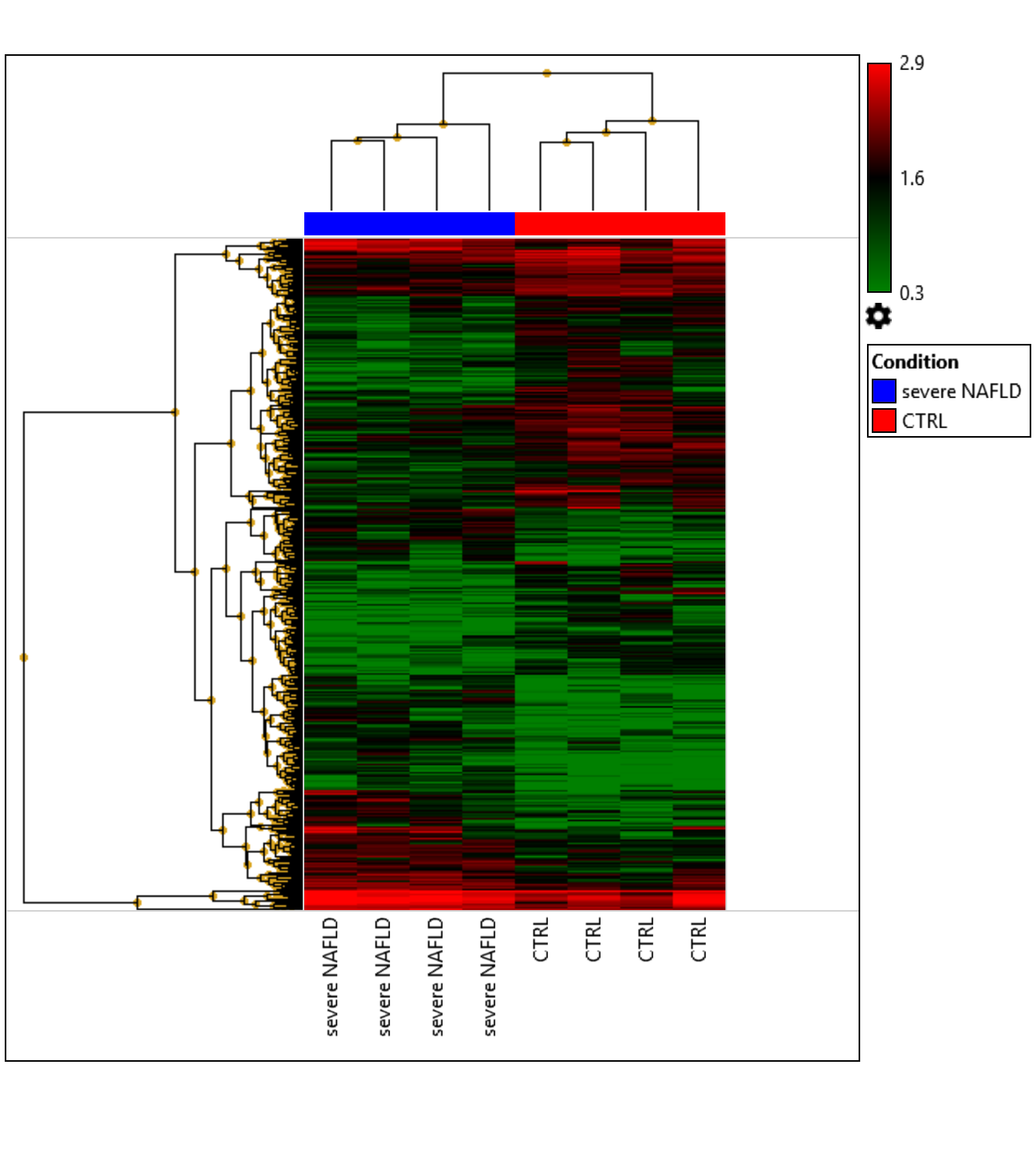

Supplement: Supplementary file 4 [file LIV-39-1742-s004.png]

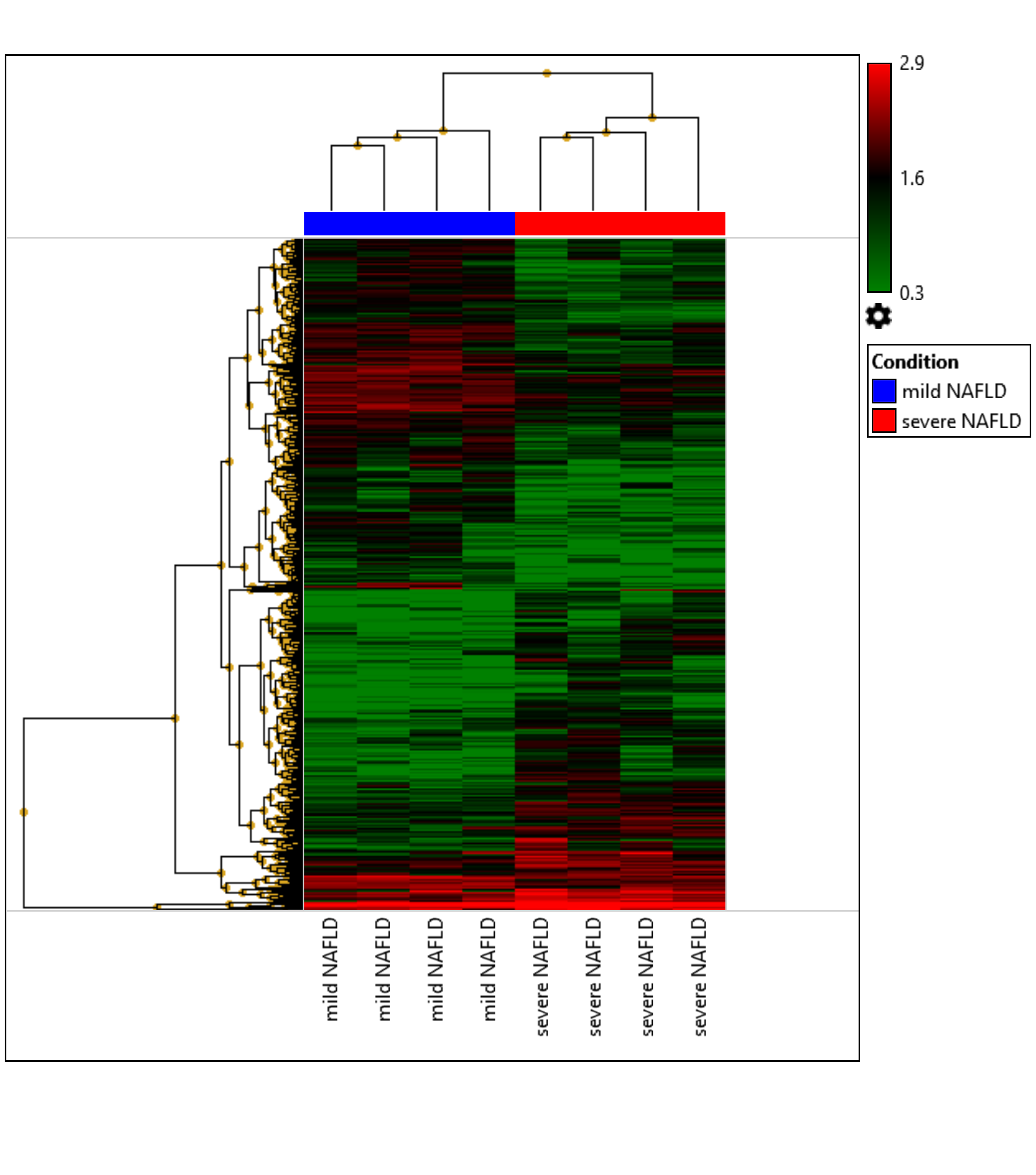

Supplement: Supplementary file 5 [file LIV-39-1742-s005.png]

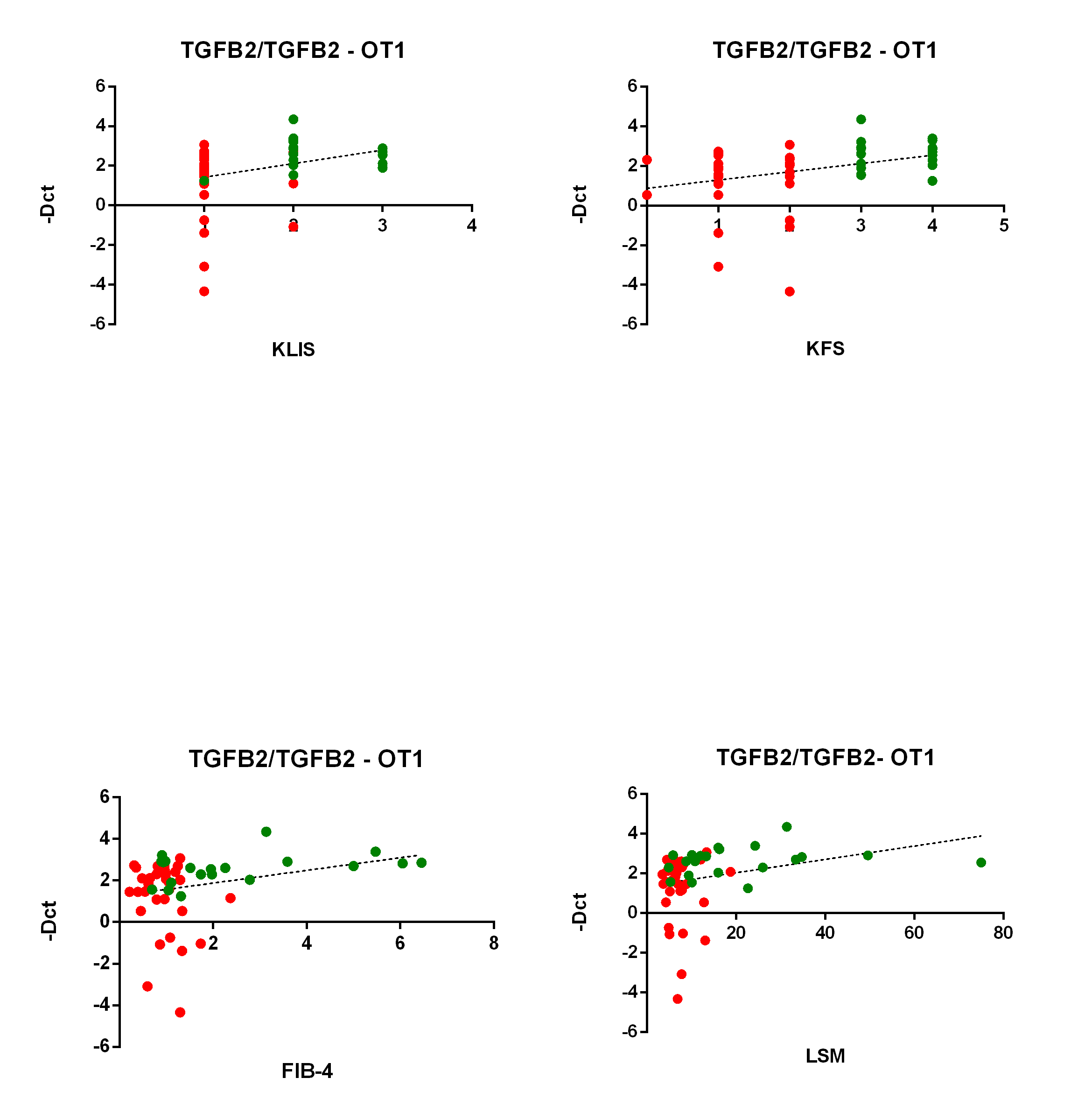

Supplement: Supplementary file 11 [file LIV-39-1742-s011.tif]
