# Supplementary material for: Serum coding and non‐coding RNAs as biomarkers of NAFLD and fibrosis severity
Source: Liver Int. 2019 Jun 26;39(9):1742–54. doi: 10.1111/liv.14167 (PMC6771597; doi:10.1111/liv.14167)
Supplement: Supplementary file 14 [file LIV-39-1742-s014.docx]

Supplementary Data

**Serum coding and non-coding RNAs as biomarkers of NAFLD and fibrosis severity**

Stefania Di Mauro^1 §^, Alessandra Scamporrino^1 §^, Salvatore Petta^2^, Francesca Urbano^1^, Agnese Filippello^1^, Marco Ragusa^3 4^, Maria Teresa Di Martino^5^, Francesca Scionti^5^, Stefania Grimaudo^2^, Rosaria Maria Pipitone^2^, Graziella Privitera^1^, Antonino Di Pino^1^, Roberto Scicali^1^, Luca Valenti^6^,Paola Dongiovanni^7^, Anna Fracanzani^7^, Agata Maria Rabuazzo^1^, Antonio Craxì^2^, Michele Purrello^3^, Francesco Purrello^1^, Salvatore Piro^1^

^§^Equal contribution

^1^ Department of Clinical and Experimental Medicine, Internal Medicine, Garibaldi-Nesima Hospital, University of Catania, Catania, Italy.

^2^ Section of Gastroenterology, Di.Bi.M.I.S., University of Palermo, Palermo, Italy.

^3^ Department of BioMedical Sciences and BioTechnology, Section of Biology and Genetics Giovanni Sichel, Unit of Molecular, Genome and Complex Systems BioMedicine, Catania, Italy.

^4^ IRCCS Associazione Oasi Maria S.S., Institute for Research on Mental Retardation and Brain Aging, Troina (EN), Italy.

^5^ Department of Experimental and Clinical Medicine, *Magna Graecia* University, Catanzaro, Italy.

^6^  Translational Medicine, University of Milan, Fondazione IRCCS Ca' Granda Pad Marangoni, Milan, Italy.

^7^ Department of Pathophysiology and Transplantation, Section of Internal Medicine, University of Milan, Fondazione Ca' Granda IRCCS Ospedale Maggiore Policlinico, Milan, Italy.

Corresponding author: Francesco Purrello, MD, Department of Clinical and Experimental Medicine, University of Catania, U.O. of Internal Medicine, Garibaldi Hospital, Via Palermo, 636 95122 Catania, Italy; Fax number: +39-0957598421; Phone number: +39-0957598401;

*E-mail: fpurrell@unict.it*

**ONLINE METHODS**

**STUDY SUBJECTS**

This study was conducted according to the Declaration of Helsinki and was approved by the ethics committee of the University Hospitals of Palermo and Milan*.* Before the procedures a written informed consent was obtained from all patients participating in this study.

This study involved two subject cohorts: a first study cohort enrolled in the Division Gastrointestinal and Liver Unit of the Palermo University Hospital including 71 patients with biopsy proven NAFLD and 29 controls and a second external cohort enrolled in Milan, IRCCS Foundation Ca' Granda Ospedale Maggiore Policlinico, which included 50 NAFLD patients. A liver biopsy was performed to confirm the diagnosis of NAFLD in patients with ultrasound findings of fatty liver and/or persistent (>6 months) elevation of ALT or AST. We excluded from our study patients with alcohol-induced or drug-induced liver disease, autoimmune or viral hepatitis and cholestatic or genetic liver disease. We also excluded patients with current or past consumption of ethanol of more than 20 g per day ([1](#_ENREF_1)). For all study participants we collected anthropometric indices (height, weight, waist circumference) and biochemical data (ALT, AST, GGT, total cholesterol, HDL cholesterol and triglycerides).

**HISTOPATHOLOGICAL EVALUATION**

For all NAFLD patients liver biopsies were obtained. The average size of liver biopsies was 25 mm (range 15-45) with at least 11 portal tracts. Liver biopsies were stained with hematoxylin and eosin and Masson’s trichrome. Histology was defined by an experienced pathologist who was blinded to patients’ clinical characteristics and defined Kleiner NAS score as the presence and severity of fibrosis ([1](#_ENREF_1), [2](#_ENREF_2)). TE was performed with the FibroScan medical device. Using the M probe (standard probe) ([3](#_ENREF_3)). A small portion of liver tissue from 12 subjects, undergoing liver biopsy for diagnostic purposes, was snap frozen in liquid nitrogen and immediately stored at -80 º C.

**BLOOD SAMPLE PROCESSING**

Blood samples were collected in separating gel vacuum collection tubes and centrifuged at 3500 rpm for 15’ at 4ºC. The upper-layer supernatant was collected and centrifuged again at 3500 rpm for 15’ at 4ºC to pellet and remove any circulating cells or debris; aliquots were stored at -80ºC until analysis.

***IN VITRO* CELL CULTURE EXPERIMENTS**

Human hepatoblastoma cell line HepG2 was purchased from ATCC (American Type Culture Collection) and cultured as previously reported ([4](#_ENREF_4)). To induce fat overloading of cells and simulate Non Alcoholic Fatty Liver Disease (NAFL) or Non Alcoholic Steato Hepatitis (NASH), HepG2 at 75% confluence were exposed to a mixture of oleate and palmitate or only to palmitate at a final fatty acid concentration of 0.5 mM for 48 h respectively ([4](#_ENREF_4)).

**RNA EXTRACTION**

Total RNA was extracted from serum samples, HepG2 cells, culture media and liver biopsies by using miRNeasy mini kit (Qiagen) according to the manufacturer's instructions ([5](#_ENREF_5)). RNA was eluted with 200 μl of Elution Solution. Subsequently, eluted RNA was precipitated with 500 μl absolute ethanol and 20 μl sodium acetate 3M overnight at -80ºC. RNA pellets were washed twice with 75% ethanol and finally resuspended in ultrapure RNase/DNase free distilled water. Total RNA was extracted from the HepG2 cell line by using TRIzol according to the manufacturer's instruction. The quantity and quality of total RNA were measured with NanoDrop (Thermo Fisher Scientific).

**MICROARRAY ANALYSIS**

Whole transcriptome analysis in serum samples of four mild NAFLD (NAS score ≤4. F=0). four severe NAFLD patients (NAS score ≥5. F=3) and four matched healthy controls was performed by using Clariom D Pico Assay (Thermo Fisher scientific) technology that detects of more than 542.500 transcripts, including mRNAs and several classes of ncRNAs (circRNAs, lncRNAs, miRNAs and their precursors, other small RNAs). 10 ng of total RNA were used to synthesize single-stranded cDNA containing T7 promoter sequence at the 5' end. 3' Adaptor was added to single-stranded cDNA as a template for double-stranded cDNA synthesis, by pre-IVT amplification reaction optimized with 12 cycles of amplification at 94°C for 30 sec. 70°C for 5 min. 4°C for 2 min. Antisense RNA (cRNA) synthesis and overnight amplification (14h) by *in vitro* transcription (IVT) of the double-stranded cDNA template, were performed using T7 RNA polymerase in a thermal cycler with a heated lid. cRNA was purified using magnetic beads and yield was assessed using a 40 μg/mL/A_260_ calculation on a NanoDrop™ ND-1000 UV-Vis Spectrophotometer. Approximately 20 μg of cRNA were obtained and then used to synthesize sense single-strand cDNA (ss-cDNA). Any remaining cRNA template was removed by RNase H digestion and ss-cDNA purification using a magnetic bead protocol. Ss-cDNA yield was evaluated using a 33 μg/mL/A_260_ calculation on a Nanodrop™ ND-1000 UV-Vis Spectrophotometer. Approximately 5.5 μg of ss-cDNA were obtained and then was fragmented using uracil DNA-glycosylase (10 U/μL) and apurinic/apyrimidinic endonuclease 1 (1.000 U/μL). Samples were then labeled with biotin using terminal deoxynucleotidyl transferase (30 U/μL). Each sample was combined with a hybridization mix with the appropriate amount of components for a 49-format array. 200 μL of the mixture were loaded into single human Clariom D arrays and placed into an Affymetrix GeneChip Hybridization Oven 645 at 45 °C and rotated at 60 rpm for 16 h. Arrays were stained using an Affymetrix GeneChip Fluidics Station 450 using the specific fluidics protocol (FS450_0001) and scanned with an Affymetrix GeneChip Scanner 3000 7G. Raw intensity CEL files generated by GeneChip™Command Console™ were imported into Transcriptome Analysis Suite (TAC) 4.0 (Applied Biosystems) and CHP files were generated for gene-level analysis.

**SINGLE REAL TIME PCR ASSAYS**

The expression of candidate coding and non-coding RNAs in serum samples (independent cohort of 88 study subjects: 63 NAFLD, 25 CTRL; external cohort of 50 NAFLD patients) liver biopsies (12 subjects: 4 CTRL, 4 mild NAFLD, 4 severe NAFLD) and HepG2 cell line samples (CTRL, OA:PA, PA in biological triplicate) was analyzed by Single Real time PCR assays by using Power SYBR Green RNA-to-CT1-Step Kit (Thermo Fisher Scientific) according to the manufacturer's protocol. Gene expression changes were calculated by applying the 2^-ΔΔCt^ method and using GAPDH as endogenous control. PCR primers were designed by using Primer Blast (*https://www.ncbi.nlm.nih.gov/tools/primer-blast/*).

**STATISTICAL ANALYSIS**

Microarray data normalization and Differentially Expressed (DE) coding and non-coding RNA identification was performed by using Transcriptome Analysis Console (TAC) Software v.4, according to the following parameters: Analysis Type: Expression Gene; Summarization Method: Gene Level - RMA. Gene-Level P-Value < 0.05 ANOVA Method: ebayes. GraphPad Prism (version 6.02) was used to: (a) perform unpaired t- test among -ΔCt values obtained after Single Real Time PCR assays; (b) perform linear regression analysis between clinical/histological data and candidate coding/non-coding RNA -ΔCt values. Receiver operating characteristics (ROC) curve analysis and multivariate logistic analyses were performed through SPSS PASW Statistics.

Supplementary Figure 1: **Fold Change distributions of Differentially Expressed Coding RNAs (A) and Non-coding RNAs** **(B)** for mild NAFLD *versus* CTRL comparison (blue bars), severe NAFLD *versus* CTRL comparison (red bars), severe NAFLD *versus* mild NAFLD comparison (green bars). Differentially expressed transcripts: p-value <0.05, Fold Change deregulation >2 and/or <-2). FC= Fold Change.

Supplementary Figure 2: Several RNA class percentage variations for each comparison: mild NAFLD *versus* CTRL, severe NAFLD *versus* CTRL, severe NAFLD *versus* mild NAFLD (as reported by TAC v.4.0 software).

Supplementary Figure 3: Hierarchical clustering of expression profile between mild NAFLD versus CTRL groups with expression fold change >2.0 or <-2 and p<0.05 (Anova Method: ebayes). Each row represents the relative expression level of a single transcript and each column represents a single analysed sample.

Supplementary Figure 4: Hierarchical clustering of expression profile between severe NAFLD versus CTRL groups with expression fold change >2.0 or <-2 and p<0.05 (Anova Method: ebayes). Each row represents the relative expression level of a single transcript and each column represents a single analysed sample.

Supplementary Figure 5: Hierarchical clustering of expression profile between and severe NAFLD versus mild NAFLD groups with expression fold change >2.0 or <-2 and p<0.05 (Anova Method: ebayes). Each row represents the relative expression level of a single transcript and each column represents a single analysed sample.

Supplementary Figure 6: **Fold Change of UBE2V1 mRNA, RP11-128N14.5 lncRNA, BNIP3L mRNA~~,~~ TGFB2/TGFB2-OT1 coding/lncRNA validated through Real Time PCRs in serum samples of patients with NAS score NAS score ≥5 respect to of patients with NAS score ≤4 (panel A)** ***n*=50: 23 NAS score ≤4, 27 NAS score ≥5. Fold Change of RP11-128N14.5 lncRNA validated through Real Time PCRs in serum samples of patients with or defined NASH respect to simple steatosis (panel B).** *n*=50: NOT NASH: 15, NASH: 35.

Supplementary Figure 7: **Fold Change deregulation of HBA2 mRNA and TGFB2/TGFB2-OT1 coding/lncRNA validated through Real Time PCRs in serum samples patients with Fibrosis Stages F=3-4 respect to F=0-2.** *n*=50 26 F=0-2, 24 F=3-4.

**Supplementary Figure 8: Linear regression relationship between serum levels of UBE2V1 and histological /biochemical data.** Histological data: Kleiner Ballooning Score (KBS), Kleiner NAS Score (KNS). Clinical data: AST, ALT, triglycerides. *n*=88: 37 F=0-2 (red), 26 F=3-4 (green), 25 CTRL (blue). Statistical significance was established at a p ≤0.05.

**Supplementary Figure 9: Linear regression relationship between serum levels of BNIP3L and histological data.** Histological data: Kleiner Lobular Inflammation Score (KLIS). Kleiner Ballooning Score (KBL). Kleiner Fibrosis Score (KFS). *n*=63: 37 F=0-2 (red) . 26 F=3-4 (green). Statistical significance was established at a p ≤0.05.

**Supplementary Figure 10: Linear regression relationship between serum levels of RP11-128N14.5 lncRNA and histological/biochemical data.** Histological data: Kleiner NAS Score (KNS). Kleiner Fibrosis Score (KFS). Clinical data: AST. *n*=88: 37 F=0-2 (red), 26 F=3-4 (green), 25 CTRL (blue). Statistical significance was established at a p ≤ 0.05.

**Supplementary Figure 11: Linear regression relationship between serum levels of TGFB2/TGFB2-OT1 and histological/biochemical data.** Histological data: Kleiner Lobular Inflammation Score (KLIF). Kleiner Fibrosis Score (KFS). Clinical data: FIB-4. Liver Stiffness Measurement (LSM) *n*=63: 37 F=0-2 (red), 26 F=3-4 (green). Statistical significance was established at a p ≤0.05.

**Supplementary Figure 12: Linear regression relationship between serum levels of HBA2 and sherveebu with biochemical data.** Clinical data: triglycerides, total cholesterol. *n*=63: 37 F=0-2 (red), 26 F=3-4 (green), 25 CTRL (blue). Statistical significance was established at a p ≤0.05.

**Supplementary Figure 13: Univariate and Multivariate ROC curve analysis for predicting APRI, FIB-4, TGFB2/TGFB2-OT1, TGFB2/TGFB2-OT1+FIB-4 as F=3-4 patient diagnosis biomarkers with respect to F=0-2 patients in external validation cohort.** APRI AUC=0.667 (95% CI=0.479-0.854 sensitivity=68.8%, specificity=66.6 %, p=9.8E-02); FIB-4 AUC=0.839 (95% CI=0.698-0.979, sensitivity=93.8%, specificity=66.7%, p=7.7E-04); TGFB2/TFB2-OT1 AUC=0.786 (95%CI=0.623-0.950, sensitivity=62.5%, specificity=94.4%, p=4.42E-03); TGFB2/TGFB2-OT1+FIB4 AUC=0.889 (95%CI=0.771-0.998, sensitivity=83.3%, specificity=83.3%, p=1.1E-04).

**Supplementary Table 1.** Anova Method eBayes p-values. Condition F-test p-values and Fold Changes values of selected coding/non-coding RNAs obtained after microarray analysis (left panel); t-test p-values and Fold Change values obtained after single Real Time PCR reactions (right panel). Statistical significance was established at a p ≤0.05. FC= Fold Change.

|  |  | **MICROARRAY DATA** | | | | | | | **REAL TIME PCR ASSAY DATA** | | | | | | | | | | | |
| --- | --- | --- | --- | --- | --- | --- | --- | --- | --- | --- | --- | --- | --- | --- | --- | --- | --- | --- | --- | --- |
|  |  | **p-value** | | |  | **Fold Change** | | | **p-value** | | | **Fold Change** | | | **p-value** | | | **Fold Change** | | |
| **GENE NAME/ SYMBOL** | **MICROARRAY PROBE** | **NAS ≤4 F0 *vs* CTRL** | **NAS ≥5 F3 *vs* CTRL** | **NAS ≤4 F0 vs NAS ≥5 F3** | **F- Test** | **NAS ≤4 F0 *vs* CTRL** | **NAS ≥5 F3 *vs* CTRL** | **NAS ≤4 F0 vs NAS ≥5 F3** | **F 0-2 *vs* CTRL** | **F 3-4 *vs* CTRL** | **F 3-4 *vs* F 0-2** | **F 0-2  *vs* CTRL** | **F 3-4  *vs* CTRL** | **F 3-4 *vs* F 0-2** | **NAS <5 *vs* CTRL** | **NAS ≥5  *vs* CTRL** | **NAS <5 *vs* NAS ≥*5*** | **NAS <5 *vs* CTRL** | **NAS ≥5  *vs* CTRL** | **NAS <5 *vs NAS* ≥*5*** |
| HBA2 | TC1600006445.hg.1 | 2.58E-01 | 8.12E-02 | **5.10E-03** | **1.67E-02** | -1.5 | 1.94 | 2.92 | 2.04E-01 | **1.08E-02** | **4.93E-02** | 16.30 | 27.39 | 18.14 | 2.07E-01 | **1.43E-02** | 7.24E-02 | 16.47 | 26.00 | 18.10 |
| UBE2V1 | TC2000010025.hg.1 | 1.84E-01 | **1.40E-03** | **1.54E-02** | **4.00E-03** | 1.55 | 3.77 | 2.43 | 7.42E-02 | **2.10E-03** | **2.69E-02** | 1.50 | 1.83 | 1.46 | 9.06E-02 | **1.90E-03** | **3.08E-02** | 1.50 | 1.80 | 1.45 |
| RP11-128N14.5 | TC1300007781.hg.1 | 5.47E-01 | **2.50E-03** | **5.40E-03** | **4.10E-03** | 1.14 | 3.64 | 3.2 | 3.41E-01 | **2.14E-02** | 8.00E-02 | 3.18 | 3.46 | 4.69 | 4.19E-01 | **1.23E-02** | **3.88E-02** | 2.80 | 3.51 | 5.06 |
| BNIP3L | TC0800007080.hg.1 | 2.29E-01 | **3.09E-02** | **1.40E-03** | **4.70E-03** | -2.12 | 2.42 | 5.14 | 2.12E-01 | **1.71E-02** | **2.37E-02** | 3.04 | 3.61 | 1.72 | 2.06E-01 | **3.04E-02** | **2.94E-02** | 2.71 | 3.94 | 1.75 |
| TGFB2/TGFB2OT1 | TC0100011621.hg.1 | 6.88E-01 | 6.39E-02 | **2.02E-02** | **4.69E-02** | 1.07 | 2.01 | 1.88 | 2.08E-01 | 6.78E-02 | **4.00E-04** | 27.21 | 49.26 | 9.20 | 3.15E-01 | 2.05E-01 | **2.87E-02** | 27.83 | 44.28 | 8.25 |
| sherveebu | TC1700007071.hg.1 | **6.00E-04** | **2.00E-04** | 4.87E-01 | **4.00E-04** | -3 | -4.67 | -1.56 | **5.30E-02** | **4.80E-02** | 5.00E-01 | -1.3 | -1.28 | -1.18 | **5.40E-02** | **3.90E-02** | 5.00E-01 | -1.3 | -1.32 | -1.19 |
|  |  |  |  |  |  |  |  |  |  |  |  |  |  |  |  |  |  |  |  |  |

Supplementary Table 2: P-values and R^2^ of linear regression analysis values between coding/non-coding RNAs and histological/biochemical data. Statistical significance was established at p ≤0.05. Kleiner Steatosis Grade (KSG). Kleiner Lobular Inflammation Score (KLIS). Kleiner Ballooning Score (KBL). Kleiner Fibrosis Score (KFS). Kleiner fibrosis stage (KFS). Liver Stiffness Measurement (LSM).

|  | **Coding RNA** | | | | | | **Non-coding RNA** | | | | **Coding/Non-coding RNA** | |
| --- | --- | --- | --- | --- | --- | --- | --- | --- | --- | --- | --- | --- |
|  | **TC2000010025.hg.1 (UBE2V1)** | | **TC1600006445.hg.1 (HBA2 )** | | **TC0800007080.hg.1 (BNIP3L)** | | **TC1300007781.hg.1 (RP11-128N14.5 )** | | **TC1700007071.hg.1 (sherveebu)** | | **TC0100011621.hg.1 (TGFB2-OT1)** | |
|  | **R square** | **p value** | **R square** | **p value** | **R square** | **p value** | **R square** | **p value** | **R square** | **p value** | **R square** | **p value** |
| **AST** | 1.48E-01 | **2.00E-03** | 3.50E-02 | 9.20E-02 | 9.00E-03 | 4.26E-01 | 2.40E-02 | **5.00E-03** | 3.20E-02 | 1.60E-01 | 4.00E-03 | 6.04E-01 |
| **ALT** | 9.70E-02 | **6.00E-03** | 0.00E+00 | 9.72E-01 | 1.00E-03 | 7.75E-01 | 1.60E-02 | 2.45E-01 | 1.80E-02 | 2.90E-01 | 2.00E-03 | 7.43E-01 |
| **GGT** | 4.00E-02 | 1.50E-01 | 5.60E-02 | 7.30E-02 | 1.00E-03 | 8.50E-01 | 0.00E+00 | 8.78E-01 | 0.00E+00 | 9.59E-01 | 7.00E-03 | 5.69E-01 |
| **TOT. CHOL** | 6.00E-03 | 5.35E-01 | 7.40E-02 | **1.80E-02** | 0.00E+00 | 8.69E-01 | 1.20E-02 | 3.49E-01 | 1.00E-03 | 8.05E-01 | 8.00E-03 | 4.65E-01 |
|  |  |  |  |  |  |  |  |  |  |  |  |  |
| **HDL** | 2.90E-02 | 2.12E-01 | 2.00E-02 | 2.77E-01 | 0.00E+00 | 9.17E-01 | 2.00E-03 | 7.15E-01 | 7.00E-03 | 5.84E-01 | 3.00E-03 | 6.74E-01 |
| **TRI** | 6.00E-02 | **4.40E-02** | 1.10E-02 | 3.72E-01 | 1.60E-02 | 3.18E-01 | 2.10E-02 | 2.23E-01 | 6.60E-02 | **3.80E-02** | 1.20E-02 | 3.87E-01 |
| **BMI** | 1.00E-02 | 3.82E-01 | 0.00E+00 | 9.56E-01 | 2.00E-02 | 2.34E-01 | 6.00E-03 | 5.03E-01 | 3.50E-02 | 1.46E-01 | 0.00E+00 | 9.78E-01 |
| **WC** | 1.40E-02 | 3.08E-01 | 2.00E-03 | 6.94E-01 | 1.80E-02 | 2.57E-01 | 4.00E-03 | 5.74E-01 | 5.00E-03 | 5.68E-01 | 1.00E-03 | 8.19E-01 |
| **PLT** | 1.00E-03 | 8.17E-01 | 5.00E-03 | 5.52E-01 | 9.00E-03 | 4.35E-01 | 4.50E-02 | 6.40E-02 | 2.10E-02 | 2.93E-01 | 1.40E-02 | 3.39E-01 |
| **KSG** | 5.60E-02 | 7.70E-02 | 1.00E-03 | 8.03E-01 | 1.50E-02 | 3.82E-01 | 1.50E-02 | 3.44E-01 | 2.00E-03 | 7.88E-01 | 1.10E-02 | 4.44E-01 |
| **KLIS** | 4.30E-02 | 1.23E-01 | 2.70E-02 | 2.03E-01 | 1.26E-01 | **8.00E-03** | 9.00E-03 | 4.51E-01 | 0.00E+00 | 9.23E-01 | 8.50E-02 | **3.10E-02** |
| **KBS** | 6.80E-02 | **5.00E-02** | 1.00E-02 | 4.44E-01 | 9.00E-02 | **2.60E-02** | 8.70E-02 | **1.90E-02** | 3.00E-03 | 7.35E-01 | 5.00E-03 | 6.09E-01 |
| **KNS** | 1.40E-01 | **4.00E-03** | 2.20E-02 | 2.53E-01 | 6.90E-02 | 5.60E-02 | 7.40E-02 | **3.10E-02** | 0.00E+00 | 9.22E-01 | 5.80E-02 | 7.70E-02 |
| **KFS** | 5.30E-02 | 8.50E-02 | 4.90E-02 | 8.40E-02 | 8.10E-02 | **3.60E-02** | 7.00E-02 | **3.70E-02** | 1.00E-03 | 7.95E-01 | 1.04E-01 | **1.60E-02** |
| **FIB-4** | 1.80E-02 | 3.15E-01 | 4.70E-02 | 9.90E-02 | 1.20E-02 | 4.42E-01 | 4.30E-02 | 1.13E-01 | 6.20E-02 | 1.21E-01 | 7.40E-02 | **4.90E-02** |
| **APRI** | 2.00E-03 | 6.34E-01 | 3.80E-02 | 1.38E-01 | 1.00E-03 | 8.22E-01 | 1.70E-02 | 3.75E-01 | 4.10E-02 | 2.02E-01 | 5.10E-02 | 1.09E-01 |
| **LSM** | 2.60E-02 | 2.38E-01 | 3.20E-02 | 1.67E-01 | 2.00E-02 | 3.04E-01 | 1.30E-02 | 3.76E-01 | 2.00E-03 | 8.15E-01 | 7.30E-02 | **4.80E-02** |

**REFERENCES**

1. YOUNES R, ROSSO C, PETTA S, et al. Usefulness of the index of NASH - ION for the diagnosis of steatohepatitis in patients with non-alcoholic fatty liver: An external validation study. Liver international : official journal of the International Association for the Study of the Liver 2017.

2. KLEINER D E, BRUNT E M, VAN NATTA M, et al. Design and validation of a histological scoring system for nonalcoholic fatty liver disease. Hepatology 2005; 41(6): 1313-21.

3. PETTA S, WONG V W, CAMMA C, et al. Improved noninvasive prediction of liver fibrosis by liver stiffness measurement in patients with nonalcoholic fatty liver disease accounting for controlled attenuation parameter values. Hepatology 2017; 65(4): 1145-55.

4. DI MAURO S, RAGUSA M, URBANO F, et al. Intracellular and extracellular miRNome deregulation in cellular models of NAFLD or NASH: Clinical implications. Nutrition, metabolism, and cardiovascular diseases : NMCD 2016; 26(12): 1129-39.

5. VALLELUNGA A, RAGUSA M, DI MAURO S, et al. Identification of circulating microRNAs for the differential diagnosis of Parkinson's disease and Multiple System Atrophy. Frontiers in cellular neuroscience 2014; 8: 156.
